# Diagnostic accuracy of warm and cold monothermal air caloric screening for bithermal canal paresis and derivation of a warm-test triage rule: a retrospective temporal validation study

**DOI:** 10.3389/fneur.2026.1953594

**Published:** 2026-10-14

**Authors:** Xiaobao Ma, Jiali Shen, Qin Zhang, Lu Wang, Wei Wang, Yulian Jin, Jun Yang, Jianyong Chen

**Affiliations:** Xinhua Hospital, Shanghai Jiao Tong University School of Medicine, Shanghai, China

**Keywords:** caloric testing, canal paresis, diagnostic accuracy, internal temporal validation, monothermal testing, spontaneous nystagmus

## Abstract

**Objective:**

To evaluate the diagnostic performance of bilateral warm (50 °C) and bilateral cold (24 °C) monothermal air caloric testing for detecting abnormal canal paresis (CP) defined by complete bithermal caloric testing, and to derive a low-risk warm-test triage rule.

**Methods:**

The primary analysis included 7,753 patients who completed all four caloric irrigations, including 5,437 in the derivation cohort and 2,316 in the temporal validation cohort. Absolute CP ≥ 25%, calculated from the peak slow-phase velocities (SPVs) of all four irrigations using a uniform formula, served as the primary reference standard. In the derivation cohort, 200 predefined combinations of warm monothermal caloric asymmetry (MCA), the lower of the two warm SPVs (WarmMin), and spontaneous nystagmus SPV were evaluated, with sensitivity ≥97.5% as the primary constraint. The selected rule was then applied unchanged to the temporal validation cohort.

**Results:**

Among 2,316 patients in the validation cohort, 846 (36.5%) had CP ≥ 25%. The AUC was 0.888 (95% CI, 0.873–0.902) for warm monothermal testing and 0.777 (95% CI, 0.756–0.798) for cold monothermal testing (AUC difference, 0.111; 95% CI, 0.084–0.136; DeLong *p* < 0.001). Of the 200 candidate rules, 99 achieved sensitivity ≥97.5%. The final candidate rule required |MCA_W| < 17.5%, WarmMin >11 °/s, and spontaneous nystagmus SPV < 4 °/s. In the validation cohort, this rule yielded a sensitivity of 98.3% (95% CI, 97.2–99.1%), specificity of 36.3% (95% CI, 33.8–38.8%), and negative predictive value of 97.4% (95% CI, 95.7–98.6%). Overall, 547 patients (23.6%) met the candidate-stop criteria, including 14 false negatives with CP ≥ 25%. Per 1,000 patients, the rule was estimated to avoid 472.4 subsequent cold irrigations while missing 6.0 cases with CP ≥ 25%. No false negatives with CP ≥ 40% were observed.

**Conclusion:**

Warm monothermal testing showed better overall discrimination than cold monothermal testing. A composite rule incorporating MCA, minimum warm response strength, and spontaneous nystagmus SPV identified a subset of low-risk patients, but still produced a small number of CP-positive false negatives. The rule should not replace complete bithermal testing and requires independent prospective validation.

## 1 Introduction

Caloric testing is a well-established method for the clinical assessment of peripheral vestibular function. Its theoretical basis can be traced to Bárány’s observation of thermally induced nystagmus (1). Irrigation of the external auditory canal with water or air above or below body temperature changes the density of endolymph in the lateral semicircular canal and generates convection, thereby eliciting the vestibulo-ocular reflex and horizontal nystagmus (2). Standard bithermal testing comprises warm and cold irrigations of both ears and uses the peak slow-phase velocity (SPV) of induced nystagmus to calculate canal paresis (CP) and directional preponderance (DP) (3). Despite the widespread use of newer tests, including video head impulse testing, caloric testing remains an important component of videonystagmographic assessment in patients with dizziness and balance disorders. It is particularly useful for evaluating asymmetry in low-frequency vestibular responses from the lateral semicircular canals (4, 5).

Complete bithermal testing nevertheless has important clinical limitations. The standard protocol requires four irrigations separated by sufficient intervals to minimize carry-over from the preceding response, and the entire examination often lasts approximately 40 min or longer. Strong thermal stimulation may provoke vertigo, nausea, or vomiting, and some patients are unable to complete the examination because of discomfort. Repeated stimulation may also increase the risk of test interruption in patients with strong vestibular responses, acute vertigo, or poor tolerance. Reducing unnecessary irrigations while retaining as much diagnostic information as possible has therefore remained an important goal of workflow optimization in vestibular laboratories (5, 6).

Since the 1960s, monothermal testing of both ears has been investigated as a means of simplifying the caloric protocol. Early studies focused on whether warm stimulation could predict the result of complete bithermal testing (7, 8). In 1971, Barber et al. (9) systematically evaluated warm monothermal screening and defined a negative screen as an interaural difference <23% with both induced nystagmus SPVs ≥11 °/s. A false negative was an examination that met these warm monothermal screening criteria but showed an abnormal result on subsequent complete bithermal testing; the reported false-negative rate was 0.7%. Subsequent studies showed that monothermal performance depends not only on interaural asymmetry, but also on absolute response strength, spontaneous nystagmus, the interaction between CP and DP, stimulus temperature, and the abnormality threshold. In 414 patients who completed bithermal testing, Lightfoot et al. (10) derived composite criteria consisting of warm monothermal caloric asymmetry (MCA) <15%, both warm responses >8 °/s, and spontaneous nystagmus <4 °/s. These criteria yielded 95% sensitivity and 71% specificity, whereas cold monothermal testing was not considered suitable for routine screening.

Subsequent studies using receiver operating characteristic (ROC) analyses, workflow efficiency measures, and alternative stimulus protocols further developed the monothermal approach (11, 12). In 645 air caloric examinations, Bush et al. (6) reported that warm monothermal air testing had a sensitivity of 87% and a negative predictive value of 90% when unilateral weakness ≥25% on complete bithermal testing defined abnormality. Warm monothermal testing could therefore screen for unilateral vestibular hypofunction, but could not predict other electronystagmographic abnormalities. A more recent study of 259 water caloric examinations also found better overall performance for warm than cold monothermal testing. Among patients without horizontal spontaneous nystagmus, warm MCA-2, calculated as (right − left) × 100/(right + left), yielded 80% sensitivity at a 25% cutoff for detecting bithermal caloric asymmetry ≥20%; spontaneous nystagmus substantially altered diagnostic performance (5).

Despite decades of investigation, the clinical role of monothermal testing remains controversial. A 2016 systematic review included 15 studies and 5,572 participants. Sensitivity ranged from 0.54 to 1.00 and specificity from 0.25 to 0.96. Warm stimulation was generally more sensitive, but the studies were heterogeneous in stimulus method, monothermal and bithermal abnormality thresholds, minimum response criteria, and patient populations. Agreement between monothermal and bithermal results was particularly limited near the boundary between normal responses and mild unilateral weakness, restricting the generalizability of fixed thresholds (13).

Previous studies have compared warm and cold monothermal testing and evaluated composite screening criteria incorporating interaural asymmetry, minimum response strength, and spontaneous nystagmus. However, most included only several hundred examinations, and evidence from large air-caloric cohorts with separate rule derivation and temporal validation remains limited. We therefore analyzed 7,753 examinations comprising bilateral 50 °C warm and bilateral 24 °C cold air irrigations.

Using derivation and temporally separated validation cohorts, we aimed to compare the ability of bilateral warm and bilateral cold monothermal testing to predict abnormal canal paresis on complete bithermal testing, derive a high-sensitivity warm-test candidate-stop rule, and evaluate the selected rule unchanged in the temporal validation cohort. We additionally examined performance across directional-preponderance strata and explored schemes pairing warm stimulation of one ear with cold stimulation of the contralateral ear. The candidate-stop rule was intended specifically to guide the testing protocol after completion of bilateral warm irrigations: when all stopping criteria were met, the two subsequent cold irrigations might be omitted; when any criterion was not met, the patient would proceed to complete bithermal testing. The rule was not intended for general clinical triage or as a replacement for complete bithermal testing when the stopping criteria were not satisfied.

## 2 Materials and methods

### 2.1 Study design and participants

This single-center retrospective diagnostic accuracy study was reported in accordance with the Standards for Reporting Diagnostic Accuracy Studies (STARD) framework (14). The source population comprised all consecutively archived caloric examinations performed in patients evaluated for dizziness or vertigo in the outpatient vestibular clinic during the study period. Common conditions in this clinical population included Ménière’s disease, vestibular migraine, recurrent episodic dizziness syndromes, and persistent postural-perceptual dizziness. Eligible examinations were required to contain the examination date, demographic information, and valid quantitative peak slow-phase velocity measurements for all four irrigations. Examinations were excluded when a complete quantitative bithermal dataset could not be established. This included examinations terminated because of physical discomfort, cardiovascular symptoms, anxiety, or patient refusal; reports containing only a qualitative response in patients with tympanic membrane perforation; examinations in which bilateral air stimulation could not be considered comparable because of middle-ear disease; and incomplete, damaged, or otherwise non-extractable reports. Under the routine laboratory protocol, patients taking vestibular-suppressant medication did not undergo caloric testing and therefore did not enter the source caloric-test records. The available archive did not permit reliable enumeration of the individual exclusion categories. The source directory contained 11,635 records. Of the 11,635 source records, 3,281 (28.2%) were excluded because a complete and reliable quantitative dataset for all four irrigations was unavailable, leaving 8,354 eligible complete examinations. A further 601 repeat examinations were excluded to preserve data independence, leaving 7,753 examinations in the primary analysis. An internal temporal split was performed according to examination date. The derivation cohort comprised 5,437 examinations performed from 14 May 2020 to 30 April 2025, and the temporal validation cohort comprised 2,316 examinations performed from 5 May 2025 to 9 July 2026 (Figure 1).

**Figure 1:**
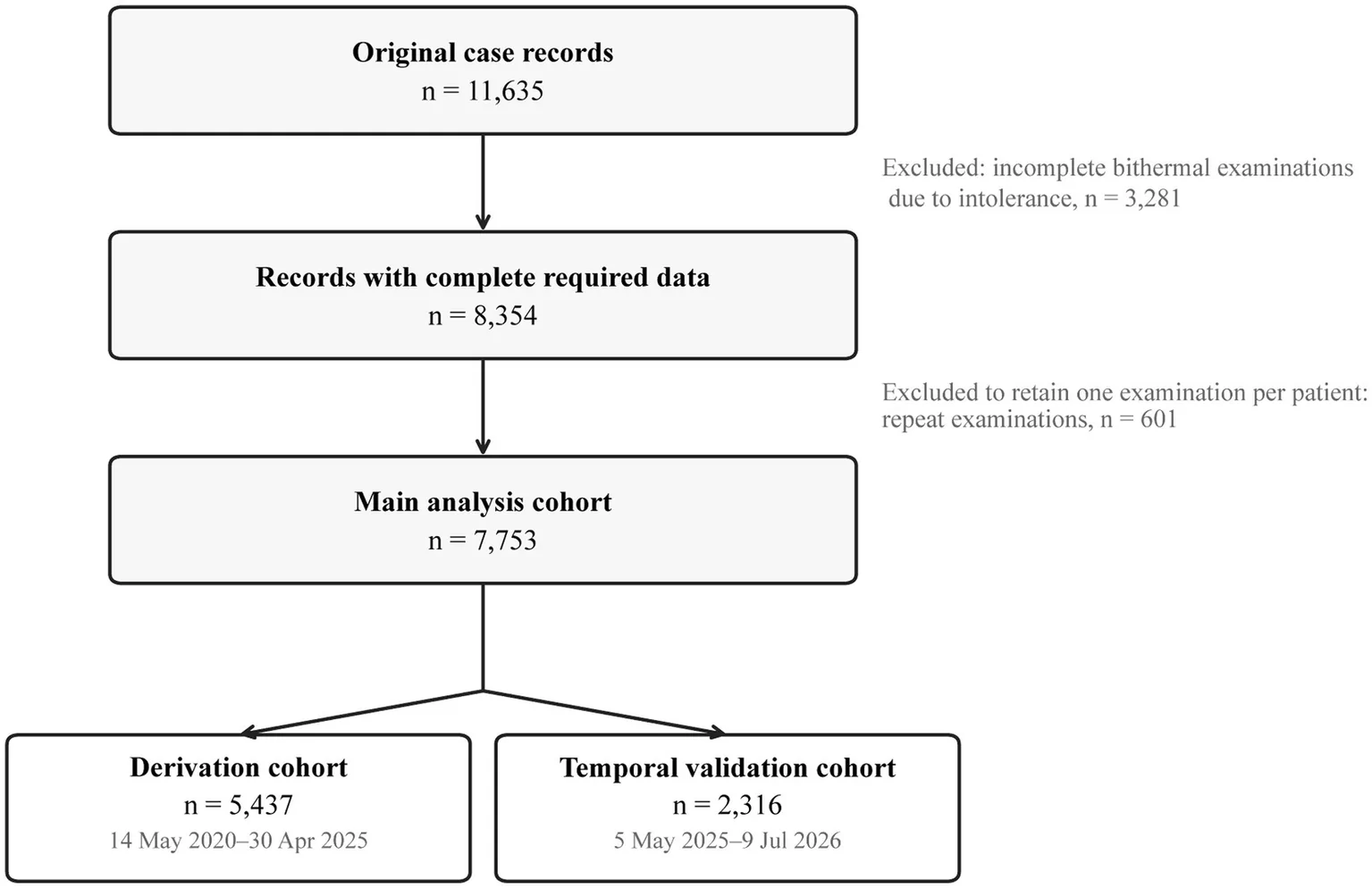
Study flow diagram. Candidate anonymous identifiers were derived from normalized name and sex because true patient identifiers were unavailable. The temporal split used complete calendar-date boundaries; no candidate identifier appeared in both cohorts. Incomplete records included examinations without a complete quantitative four-irrigation dataset because testing was terminated owing to discomfort, cardiovascular symptoms, anxiety, or refusal; only qualitative responses were available; bilateral air stimulation was not comparable because of middle-ear disease; or the archived report was incomplete, damaged, or non-extractable. Category-specific exclusion counts were unavailable.

Because unique hospital patient identifiers were unavailable in the extracted dataset, a candidate patient identifier was constructed from the combination of the standardized Chinese name and sex. All names were recorded in Chinese characters, and records were linked only when both the name and sex matched exactly; no phonetic or fuzzy name matching was performed. For candidate identifiers associated with more than one complete examination, only the earliest eligible examination was retained. Deduplication was completed before temporal cohort allocation, ensuring that no identical candidate identifier was included in both the derivation and temporal validation cohorts.

### 2.2 Caloric test equipment and protocol

Caloric testing was performed in a darkened room. Eye movements were recorded using a VO425 videonystagmography (VNG) system (Interacoustics, Denmark), and air irrigations were delivered with an Air Fx caloric irrigator from the same manufacturer. Participants lay supine with the head elevated by approximately 30° to orient the horizontal semicircular canals close to the vertical plane, and wore light-excluding VNG goggles. They were instructed to gaze straight ahead, keep their eyes open, and minimize blinking throughout the examination. The four air irrigations were delivered in a fixed warm-before-cold and right-before-left sequence: right ear at 50 °C, left ear at 50 °C, right ear at 24 °C, and left ear at 24 °C. This sequence reflected the routine clinical protocol and was neither randomized nor counterbalanced. Consequently, stimulus temperature was inseparable from its position in the testing sequence, with both warm irrigations always preceding both cold irrigations. Airflow was 8 L/min and each irrigation lasted 60 s, corresponding to an approximate delivered volume of 8 L. The nozzle was directed toward the tympanic membrane and held stably as deep as practicable without contacting the membrane or occluding the external auditory canal. Successive irrigations were separated by 5 min, and testing proceeded only after the induced nystagmus had resolved and vertigo had subsided (15, 16). Immediately before the first caloric irrigation, spontaneous nystagmus was recorded for 60 s with the participant in the same supine position with the head elevated by approximately 30° as used for caloric testing. The VNG system identified the slow and fast phases of nystagmus in real time and automatically determined the nystagmus direction and SPV; the recordings were subsequently reviewed manually. The absolute SPV magnitude was used in the candidate-stop rule and stratified analyses, whereas the direction was retained separately as left-beating or right-beating. When spontaneous nystagmus was present, the VNG system applied direction-dependent baseline correction to each of the four caloric responses. When the spontaneous and caloric-induced nystagmus were in the same direction, the spontaneous-nystagmus component was subtracted from the observed response; when they were in opposite directions, its magnitude was added. The resulting corrected RW, LW, RC, and LC values were used in all subsequent calculations. For each irrigation, the software automatically identified the analysis segment with the highest mean SPV. The automatically selected segments and corresponding peak-SPV values were reviewed by two experienced vestibular technicians, both of whom held hospital-recognized intermediate professional titles. The review determined whether the selected segment represented a sustained caloric response rather than an isolated outlying value or recording artifact. If an outlying value or artifact was incorrectly identified as the peak response, the curve was re-examined and an artifact-free segment representing the highest sustained mean SPV was manually selected. Examinations for which reliable peak-SPV values could not be obtained for all four irrigations after review were considered incomplete and were excluded from the analysis.

### 2.3 Outcome measures and reference standard

The four air irrigations were right warm at 50 °C (RW), left warm at 50 °C (LW), right cold at 24 °C (RC), and left cold at 24 °C (LC); peak induced nystagmus SPV was used for each response (17, 18). When spontaneous nystagmus was present, RW, LW, RC, and LC referred to the direction-corrected peak-SPV values generated by the VNG system as described above. These corrected values were used to calculate CP, DP, warm and cold MCA, WarmMin, and ColdMin; no additional correction was applied directly to these derived indices after calculation. Absolute CP was calculated uniformly as CP = |(RW + RC) − (LW + LC)|/(RW + LW + RC + LC) × 100% (19, 20). The primary reference standard was CP ≥ 25%, with CP ≥ 20%, ≥30%, and ≥40% used as sensitivity-analysis endpoints (21). Because monothermal MCA and bithermal CP were calculated from overlapping caloric responses, the estimand was prediction of the dichotomized bithermal CP classification rather than diagnosis against an independent measure of vestibular dysfunction. Uniform recalculation ensured that the same reference-standard formula was applied to every examination. Signed warm MCA was defined as MCA_W = (RW − LW)/(RW + LW) × 100% (5, 22), and the index test used |MCA_W|. WarmMin = min(RW, LW) and WarmTotal = RW + LW. The corresponding cold measures were MCA_C = (RC − LC)/(RC + LC) × 100%, ColdMin = min(RC, LC), and ColdTotal = RC + LC (10). Absolute DP was calculated as |(RW + LC) − (LW + RC)|/(RW + LW + RC + LC) × 100% (19, 23).

The weaker side on complete bithermal testing was determined from the sign of signed CP. The weaker side on warm and cold monothermal testing was the ear with the lower SPV at the corresponding temperature. Equal bilateral responses at the corresponding monothermal temperature (RW = LW for warm testing or RC = LC for cold testing) were classified as having an indeterminate monothermal weaker side and were excluded from the temperature-specific weak-side agreement analysis. The numbers included and excluded were reported separately for each cohort and temperature. In the original clinical reports, absence of spontaneous nystagmus was explicitly documented as negative (“−”) and was coded as 0 °/s for analysis; when spontaneous nystagmus was present, the reported SPV was retained. Stratified analyses used four SPV categories: 0, >0 to <2, 2 to <4, and ≥4 °/s (5, 10).

### 2.4 Derivation of monothermal candidate rules

The derivation cohort was used to evaluate a predefined candidate grid comprising 10 warm MCA thresholds (5, 7.5, 10, 12.5, 15, 17.5, 20, 22.5, 25, and 30%), five WarmMin thresholds (>5, >6, >8, >10, and >11 °/s), and four spontaneous-nystagmus conditions (SPV = 0, <2, <4, and ≤4 °/s), yielding 200 distinct parameter combinations. The candidate values were selected to span thresholds previously used in monothermal screening and additional intermediate values needed to characterize the safety–efficiency relationship. The MCA grid included stringent screening thresholds and values surrounding previously reported cutoffs of approximately 10, 15, 23, and 25% (9–12). The WarmMin grid included the previously reported minimum-response criteria of 8 and 11 °/s, together with adjacent lower and intermediate values (9, 10). The spontaneous-nystagmus conditions ranged from complete absence to progressively more permissive limits, including the 4 °/s boundary used in the British Society of Audiology (BSA) recommended procedure (10, 24). A patient met the candidate-stop criteria only when all three conditions were satisfied; otherwise, complete bithermal testing was required. For contextual comparison, the BSA-like candidate-stop criteria were operationally defined as absolute warm MCA < 15%, both warm responses >8 °/s, and spontaneous nystagmus SPV ≤ 4 °/s, following Appendix C of the 2010 British Society of Audiology recommended procedure. The term “BSA-like” was used because the retrospective dataset permitted implementation of these numerical thresholds but did not reproduce all procedural and clinical decision elements of the complete BSA recommendation. This comparison was conducted *post hoc* after validation-cohort results had been examined and was not used to select or retune the final rule.

Before validation-cohort results were reviewed, the initial analysis plan specified evaluation of candidate sets meeting sensitivity targets of ≥95%, ≥97.5%, and ≥99%, with priority given to high sensitivity. During preliminary analyses, the investigators reviewed the validation-cohort AUCs, performance at individual MCA thresholds, and the performance and false-negative distributions of earlier candidate rules. The expanded spontaneous-nystagmus grid, designation of ≥97.5% as the principal working constraint, the 0.5-percentage-point plateau, the ordered tie-breaking criteria, and some reporting decisions were finalized subsequently during refinement of the derivation-stage analysis. The ≥97.5% threshold was an investigator-defined pragmatic target intended to limit the derivation-stage false-negative proportion among patients with CP ≥ 25% to no more than 2.5%. It was not based on a guideline-endorsed threshold or a formal noninferiority margin. Combinations with a derivation-cohort sensitivity point estimate <97.5% were excluded, and the remaining rules were ranked by specificity. Rules with specificity within 0.5 percentage points of the highest observed value were considered to lie on a performance plateau and were further compared, in order, according to the numbers of false negatives with CP ≥ 40% and CP ≥ 30%, followed by sensitivity, negative predictive value, candidate-stop rate, and rule simplicity. The sensitivity point estimate was used as an empirical filtering and ranking criterion rather than as a confidence-bound requirement. The final 200-rule ranking and numerical threshold selection were performed using the derivation cohort alone; validation-cohort metrics were not entered into the formal ranking algorithm or used to numerically retune the selected rule.

The analytical framework was not prospectively registered. These values are therefore described as predefined candidate thresholds rather than prospectively prespecified thresholds. To evaluate the incremental value of WarmMin and spontaneous nystagmus, a warm-MCA-only rule was selected independently in the derivation cohort. Among the prespecified warm MCA thresholds, the threshold with the highest specificity subject to the same sensitivity constraint of ≥97.5% was selected and then applied unchanged to the temporal validation cohort. The warm-MCA-only rule and the final composite rule were compared using paired patient-level analyses of sensitivity, specificity, NPV, candidate-stop rate, and missed CP ≥ 25% cases. This comparison was not used to modify the final composite rule.

Requiring complete bithermal testing was defined as an index-test-positive result. Accordingly, a true positive (TP) was a CP-positive patient for whom the rule required complete testing; a false negative (FN) was a CP-positive patient whom the rule classified as a candidate stop; a true negative (TN) was a CP-negative patient classified as a candidate stop; and a false positive (FP) was a CP-negative patient for whom the rule required complete testing.

### 2.5 Additional analyses

The area under the receiver operating characteristic curve (AUC), together with performance at the 5% MCA threshold, was assessed for warm and cold monothermal testing within absolute DP strata of <10, 10 to <20%, 20 to <30%, and ≥30%, and within the four spontaneous-nystagmus SPV strata. Because DP is available only after complete bithermal testing, it was used to explain variation in monothermal performance and was not included in the clinical candidate-stop rule.

Two exploratory cross-temperature schemes were evaluated: RW + LC and LW + RC. To account for the overall difference in response strength between warm and cold stimulation, the median WarmTotal/ColdTotal ratio, k, was estimated in the derivation subgroup with CP < 20%, DP < 10%, and spontaneous nystagmus SPV = 0. The indices |(RW − k × LC)/(RW + k × LC)| and |(LW − k × RC)/(LW + k × RC)| were then calculated, and the fixed coefficient and derivation-cohort thresholds were applied unchanged to the validation cohort. Because stimulus temperature and ear side were completely confounded, these analyses were considered exploratory.

### 2.6 Statistical analysis

Continuous variables were summarized as mean (standard deviation) or median [interquartile range], as appropriate, and categorical variables as n (%). Baseline differences between the derivation and validation cohorts were described primarily using standardized mean differences (SMDs), avoiding sole reliance on *p* values in this large sample. Diagnostic measures included the AUC, sensitivity, specificity, positive predictive value (PPV), negative predictive value (NPV), positive and negative likelihood ratios (LR + and LR−), false-negative rate, false-positive rate, accuracy, candidate-stop rate, weak-side agreement, and Cohen’s κ (25). Exact Clopper–Pearson 95% confidence intervals (CIs) were used for binomial proportions, and log-method 95% CIs were used for likelihood ratios; non-estimable quantities were reported as such. AUCs, AUC differences, paired differences, and per-1,000 efficiency measures were estimated with 2,000 bootstrap resamples. Because the primary analysis retained only one examination per candidate patient, patient-level resampling used the candidate patient as the sampling unit.

Correlated ROC curves for warm and cold testing were compared using DeLong’s test (26). Sensitivity and specificity at the 5% MCA threshold, and for the final rule versus the BSA-like criteria, were compared using the exact paired McNemar test; 95% CIs for paired differences were obtained by bootstrap resampling (24, 27). Uncertainty for zero-event results was described using an exact binomial upper bound. A fixed random seed of 20,260,717 was used for all stochastic procedures. Analyses were performed in Python 3.12 using pandas, NumPy, SciPy, scikit-learn, and matplotlib. All formulas, thresholds, and exclusion rules were documented in independently executable scripts.

### 2.7 Use of generative artificial intelligence

OpenAI Codex (desktop application, version 26.721.41059; GPT-5.6 Sol mode; OpenAI), a generative artificial intelligence (AI) tool, was used to assist with Python code drafting and debugging, the preparation and formatting of data-derived figures and tables, English translation, and language editing. All data-processing decisions, variable definitions, statistical analyses, numerical results, citations and references, and figure and table contents were checked by the authors against the source data, analysis scripts, and output files. All AI-assisted content was critically reviewed, revised, and approved by the authors. The authors also checked the AI-assisted written and visual content for factual accuracy and plagiarism and take full responsibility for the accuracy and integrity of the manuscript.

## 3 Results

### 3.1 Study population and baseline characteristics

The primary analysis included 7,753 patients, with 5,437 in the derivation cohort and 2,316 in the temporal validation cohort. Age ranged from 3 to 94 years. A total of 1,846 patients (23.8%) were younger than 18 years, including 1,253 of 5,437 patients (23.0%) in the derivation cohort and 593 of 2,316 patients (25.6%) in the temporal validation cohort. CP ≥ 25% was present in 35.9 and 36.5% of the derivation and validation cohorts, respectively; women accounted for 60.5 and 59.4%, respectively (Table 1; Supplementary Figure S1). The proportion of examinations with spontaneous nystagmus SPV of 0 °/s was lower in the temporal validation cohort than in the derivation cohort (38.0% vs. 48.2%, SMD = 0.207). However, the proportion satisfying the spontaneous-nystagmus component of the final candidate rule (SPV < 4 °/s) was similar between the temporal validation and derivation cohorts (95.9% vs. 96.7%, SMD = 0.041), indicating that the temporal shift occurred predominantly among the lower SPV categories rather than across the final rule threshold.

**Table 1:** Baseline characteristics of the derivation and temporal validation cohorts.

Characteristic	Derivation cohort	Temporal validation cohort	Standardized mean difference
Participants, *n*	5,437	2,316	—
Age range, years	3–94	3–94	—
Age <18 years, *n* (%)	1,253 (23.0%)	593 (25.6%)	−0.060
Age, years, mean (SD)	45.5 (23.9)	44.4 (24.5)	0.044
Female sex, *n* (%)	3,288 (60.5%)	1,376 (59.4%)	0.022
CP ≥ 25%, *n* (%)	1,952 (35.9%)	846 (36.5%)	−0.013
DP ≥ 20%, *n* (%)	1,783 (32.8%)	771 (33.3%)	−0.011
Right warm SPV, °/s, median [IQR]	15.0 [8.0–27.0]	16.0 [9.0–27.0]	−0.044
Left warm SPV, °/s, median [IQR]	16.0 [9.0–29.0]	18.0 [10.0–31.0]	−0.103
Right cold SPV, °/s, median [IQR]	13.0 [8.0–19.0]	13.0 [8.0–21.0]	−0.098
Left cold SPV, °/s, median [IQR]	13.0 [8.0–21.0]	15.0 [9.0–23.0]	−0.138
Warm total SPV, °/s, median [IQR]	34.0 [20.0–55.0]	36.0 [22.0–58.0]	−0.086
Cold total SPV, °/s, median [IQR]	27.0 [18.0–39.0]	29.0 [19.0–42.0]	−0.137
Spontaneous nystagmus SPV, °/s, median [IQR]	1.0 [0.0–1.0]	1.0 [0.0–1.0]	−0.121
Spontaneous nystagmus SPV < 4 °/s, *n* (%)	5,256 (96.7%)	2,221 (95.9%)	0.041
Spontaneous nystagmus SPV = 0°/s, *n* (%)	2,622 (48.2%)	881 (38.0%)	0.207
Spontaneous nystagmus SPV > 0 to <2°/s, *n* (%)	1,676 (30.8%)	901 (38.9%)	−0.170
Spontaneous nystagmus SPV 2 to <4°/s, *n* (%)	958 (17.6%)	439 (19.0%)	−0.035
Spontaneous nystagmus SPV ≥ 4°/s, *n* (%)	181 (3.3%)	95 (4.1%)	−0.041

Values are mean (SD), median [IQR], median [range], or *n* (%), as specified.

### 3.2 Overall discrimination of warm and cold monothermal testing

In the validation cohort, the AUC was 0.888 (95% CI, 0.873–0.902) for warm MCA and 0.777 (95% CI, 0.756–0.798) for cold MCA. The AUC difference was 0.111 (95% CI, 0.084–0.136, DeLong z = 8.30, *p* < 0.001), indicating better discrimination for warm MCA than for cold MCA within the fixed warm-before-cold protocol evaluated in this study (Figure 2; Supplementary Figures S2–S3).

**Figure 2:**
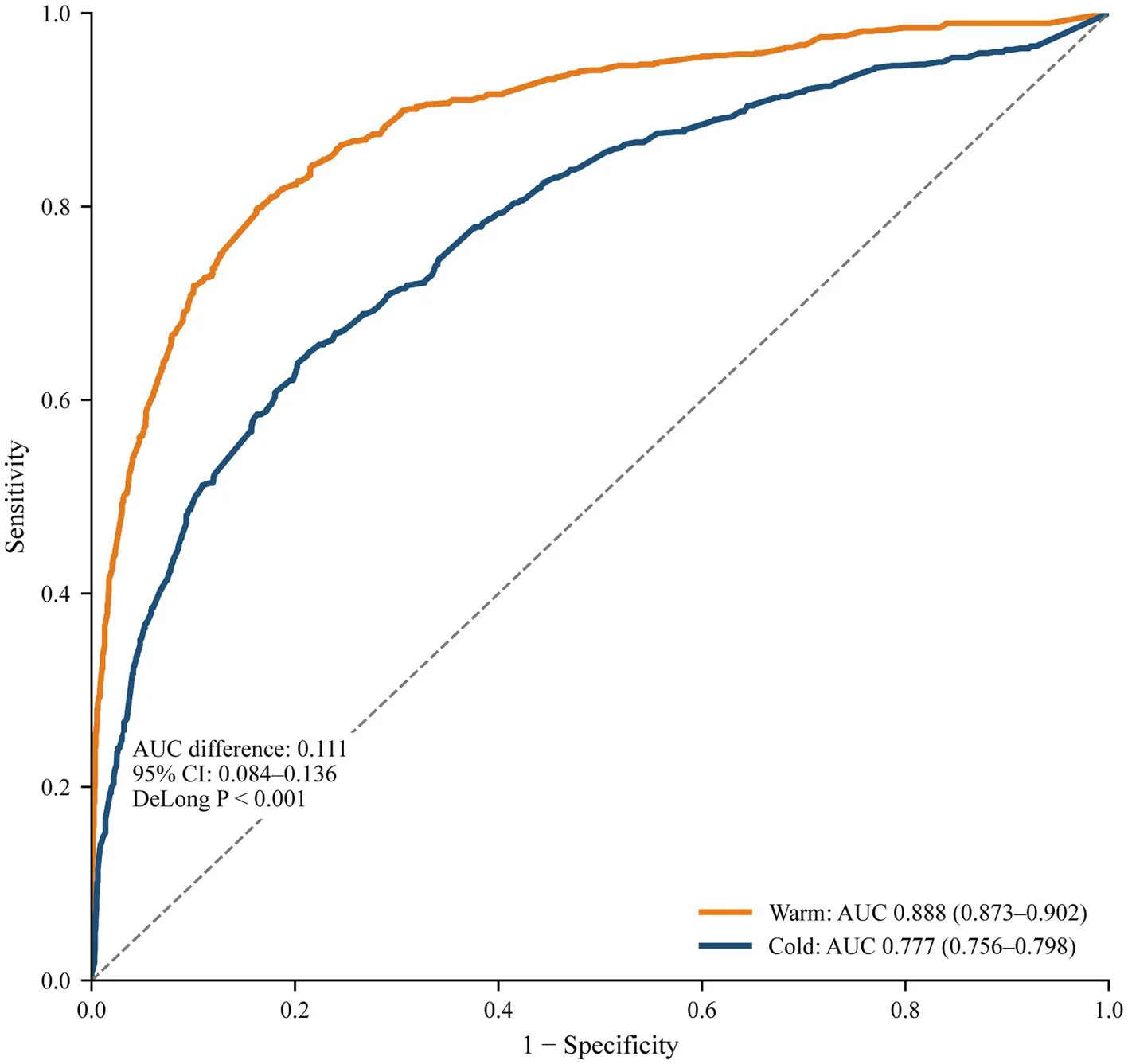
Receiver operating characteristic curves for absolute warm and cold MCA against CP ≥ 25% in the temporal validation cohort (*n* = 2,316). AUC confidence intervals and the paired AUC difference were estimated by 2,000 stratified bootstrap samples; correlated AUCs were compared using DeLong’s test (*z* = 8.30, *p* < 0.001).

At the 5% single-MCA threshold, sensitivity in the validation cohort was 98.9% (95% CI, 98.0–99.5%) for warm testing and 94.7% (95% CI, 92.9–96.1%) for cold testing. The paired difference was 4.3 percentage points (95% CI, 2.7–5.9; McNemar *p* < 0.001). Specificity was 15.6% for warm testing and 18.2% for cold testing, a difference of −2.7 percentage points (95% CI, −5.2 to 0.1; *p* = 0.058). Performance across all evaluated warm and cold MCA thresholds in the derivation and temporal validation cohorts is reported in Supplementary Table S1. Among the 846 CP-positive examinations in the validation cohort, the weaker side on complete bithermal testing was determinable in all cases. For warm testing, nine examinations had equal bilateral warm responses and were excluded, leaving 837 examinations for analysis; the monothermal and bithermal weaker sides agreed in 831/837 examinations (99.3, 95% CI, 98.4–99.7%; Cohen’s κ = 0.985, 95% CI, 0.972–0.995). For cold testing, 29 examinations had equal bilateral cold responses and were excluded, leaving 817 examinations; agreement was observed in 767/817 examinations (93.9, 95% CI, 92.0–95.4%; Cohen’s κ = 0.872, 95% CI, 0.837–0.905) (Table 2).

**Table 2:** Diagnostic performance of the 5% single-MCA threshold.

Cohort	Scheme	AUC (95% CI)	Sensitivity (95% CI)	Specificity (95% CI)	NPV (95% CI)	LR − (95% CI)	Candidate stop rate (95% CI)	Weak-side agreement, *n*/*N* (%) (95% CI)	Weak-side Cohen κ (95% CI)
Derivation cohort	Warm	0.871 (0.861–0.881)	98.2% (97.5–98.7%)	17.8% (16.6–19.2%)	94.5% (92.5–96.1%)	0.103 (0.074–0.144)	12.1% (11.2–13.0%)	1,910/1,930 (99.0%; 98.4–99.4%)	0.979 (0.969–0.987)
Derivation cohort	Cold	0.783 (0.770–0.795)	95.2% (94.1–96.1%)	18.5% (17.2–19.8%)	87.3% (84.6–89.6%)	0.261 (0.211–0.321)	13.6% (12.7–14.5%)	1,760/1,894 (92.9%; 91.7–94.0%)	0.856 (0.832–0.878)
Temporal validation cohort	Warm	0.888 (0.873–0.902)	98.9% (98.0–99.5%)	15.6% (13.8–17.5%)	96.2% (92.9–98.3%)	0.068 (0.035–0.132)	10.3% (9.1–11.6%)	831/837 (99.3%; 98.4–99.7%)	0.985 (0.972–0.995)
Temporal validation cohort	Cold	0.777 (0.756–0.798)	94.7% (92.9–96.1%)	18.2% (16.3–20.3%)	85.6% (81.2–89.3%)	0.292 (0.215–0.395)	13.5% (12.1–15.0%)	767/817 (93.9%; 92.0–95.4%)	0.872 (0.837–0.905)

Weak-side agreement and Cohen’s κ were calculated only among examinations with CP ≥25% in which both the reference-standard and monothermal weaker sides were determinable. The reference-standard weaker side was determinable in all CP-positive examinations. In the derivation cohort, 22 warm ties and 58 cold ties were excluded, leaving 1,930 and 1,894 examinations, respectively. In the temporal validation cohort, nine warm ties and 29 cold ties were excluded, leaving 837 and 817 examinations, respectively. A tie was defined as RW = LW for warm testing or RC = LC for cold testing.

### 3.3 Screening of 200 warm composite rules

Of the 200 predefined candidate combinations, 152 achieved sensitivity ≥95%, 99 achieved ≥97.5%, and 31 achieved ≥99%. Under the primary sensitivity constraint, the rule with the highest specificity required |MCA_W| < 17.5%, WarmMin >11 °/s, and spontaneous nystagmus SPV ≤ 4 °/s, yielding a specificity of 37.1%. The otherwise identical rule using SPV < 4 °/s had a specificity of 36.8%, only 0.3 percentage points lower and within the investigator-defined 0.5-percentage-point plateau. The two rules had identical numbers of false negatives with CP ≥ 30% and CP ≥ 40%, whereas the SPV < 4 °/s rule had slightly higher sensitivity and NPV. It was therefore selected as the final candidate rule (Figure 3; Supplementary Figure S4; Supplementary Table S4). Full derivation-cohort results for all 200 candidate rules are provided in Supplementary Table S2.

**Figure 3:**
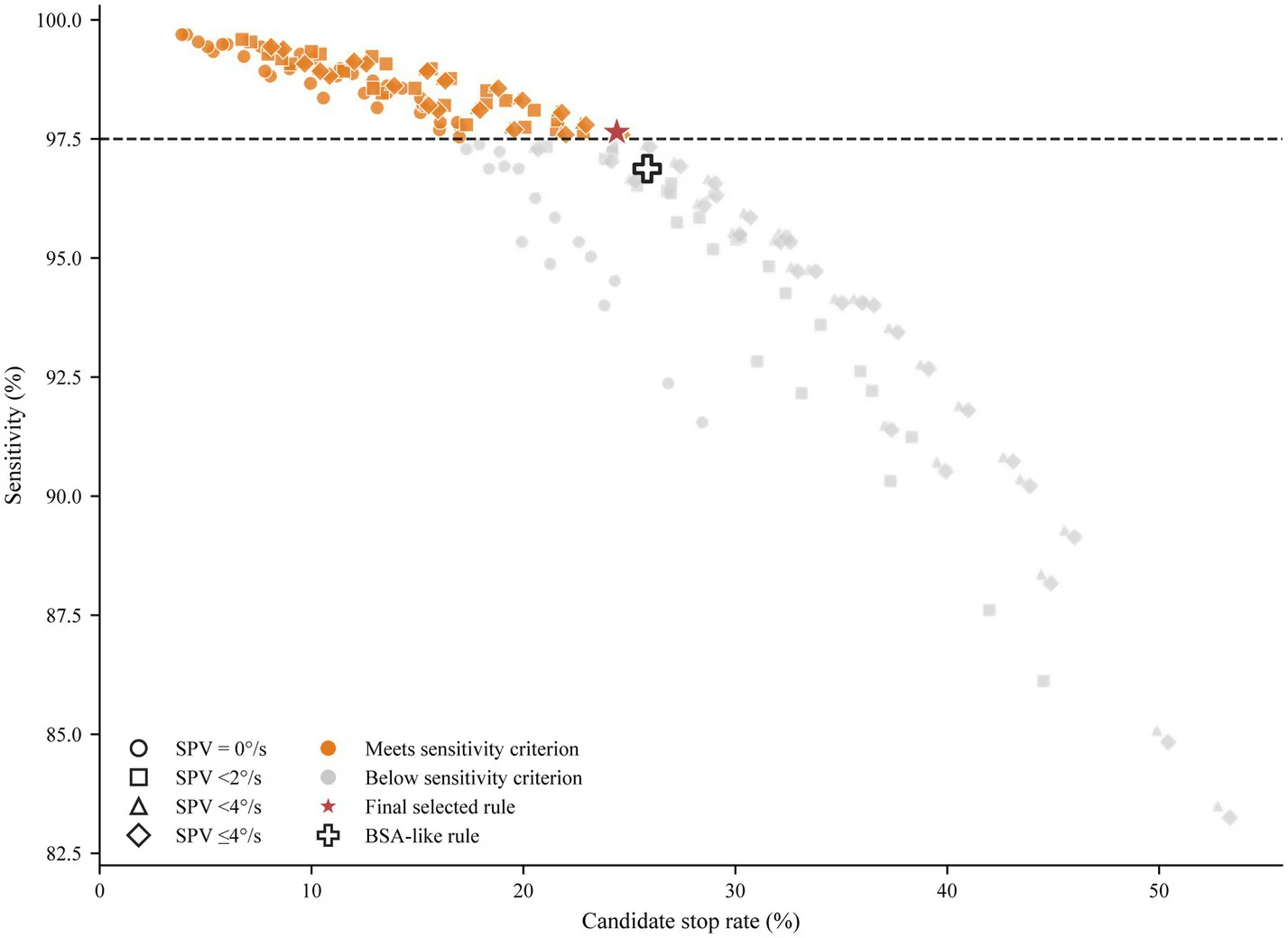
Safety–efficiency frontier for all 200 predefined candidate warm composite rules in the derivation cohort (*n* = 5,437). Each point represents one distinct combination of 10 MCA thresholds, five minimum warm SPV thresholds, and four spontaneous nystagmus SPV conditions. Orange points met the investigator-defined sensitivity criterion of 97.5%; grey points did not. The final selected rule and the BSA-like criteria are highlighted. Validation-cohort metrics were not entered into the formal rule-ranking algorithm or used to numerically retune the selected rule.

### 3.4 Derivation and temporal validation of the final candidate rule

In the derivation cohort, the final candidate rule yielded TP/FN/TN/FP counts of 1,906/46/1,281/2,204. Sensitivity was 97.6% (95% CI, 96.9–98.3%), specificity was 36.8% (95% CI, 35.2–38.4%), NPV was 96.5% (95% CI, 95.4–97.5%), and the candidate stop rate was 24.4% (95% CI, 23.3–25.6%).

When applied unchanged to the validation cohort, the rule yielded TP/FN/TN/FP counts of 832/14/533/937. Sensitivity was 98.3% (95% CI, 97.2–99.1%), specificity was 36.3% (95% CI, 33.8–38.8%), positive predictive value was 47.0% (95% CI, 44.7–49.4%), NPV was 97.4% (95% CI, 95.7–98.6%), LR + was 1.543 (95% CI, 1.483–1.605), and LR − was 0.046 (95% CI, 0.027–0.077). The false-negative rate was 1.7% (95% CI, 0.9–2.8%), accuracy was 58.9% (95% CI, 56.9–61.0%), and the candidate stop rate was 23.6% (95% CI, 21.9–25.4%) (Table 3; Supplementary Table S5; Figure S5).

**Table 3:** Derivation and temporal validation performance of the final warm composite rule.

Cohort	*n*	TP	FN	TN	FP	Sensitivity (95% CI)	Specificity (95% CI)	PPV (95% CI)	NPV (95% CI)	LR − (95% CI)	Candidate stop rate (95% CI)
Derivation cohort	5,437	1,906	46	1,281	2,204	97.6% (96.9–98.3%)	36.8% (35.2–38.4%)	46.4% (44.8–47.9%)	96.5% (95.4–97.5%)	0.064 (0.048–0.086)	24.4% (23.3–25.6%)
Temporal validation cohort	2,316	832	14	533	937	98.3% (97.2–99.1%)	36.3% (33.8–38.8%)	47.0% (44.7–49.4%)	97.4% (95.7–98.6%)	0.046 (0.027–0.077)	23.6% (21.9–25.4%)

Final composite rule: absolute warm MCA <17.5%, both warm SPVs >11 °/s, and spontaneous nystagmus SPV <4 °/s. The validation cohort was not used for rule selection.

### 3.5 Estimated irrigation reduction and false-negative burden

In a retrospective simulation of the validation cohort, 547 of 2,316 patients (23.6%) would have met the candidate-stop criteria, whereas 1,769 would have required complete bithermal testing. Had the rule been applied prospectively, this would have corresponded to an estimated reduction of 472.4 cold irrigations per 1,000 patients (bootstrap 95% CI, 437.8–507.8), representing 23.6% of planned cold irrigations and 11.8% of all planned warm and cold irrigations. The estimated numbers of missed cases per 1,000 patients were 6.0 for CP ≥ 25% (95% CI, 3.0–9.5) and 1.7 for CP ≥ 30% (95% CI, 0.4–3.5). These estimates were derived retrospectively; actual examination time, tolerability, test completion, and adverse events were not evaluated (Figure 4; Supplementary Table S6).

**Figure 4:**
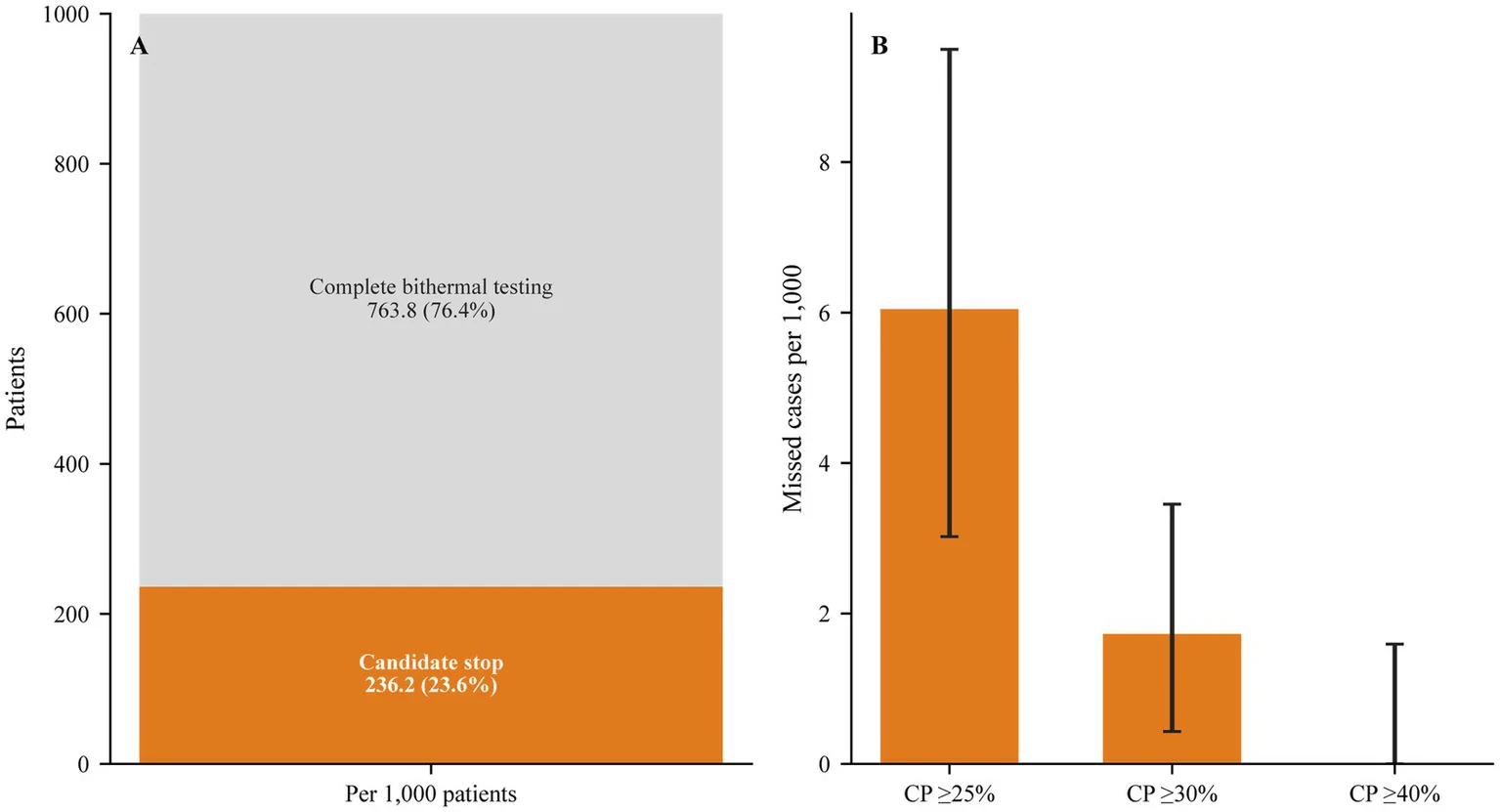
Validation-cohort efficiency simulation for the final warm composite rule. **A** Expected numbers of candidate-stop and complete-bithermal-testing patients per 1,000. Each candidate stop avoids two planned cold irrigations. **B** Missed CP ≥ 25%, CP ≥ 30%, and CP ≥ 40% cases per 1,000. Bootstrap 95% confidence intervals are shown for non-zero burdens; the zero CP ≥ 40% bar uses the exact binomial upper bound. Among 439 validation patients with CP ≥ 40%, 0 false negatives were observed (exact 95% false-negative-rate upper bound 0.84%).

Among the 14 false negatives with CP ≥ 25%, 10 had CP from 25 to <30% and four had CP from 30 to <40%; none had CP ≥ 40%. There were 439 patients with CP ≥ 40% in the validation cohort, and 0/439 corresponded to an exact 95% upper bound of 0.84% for the false-negative rate. A zero observed event count does not imply that the true risk is zero. Because the final rule itself required WarmMin >11 °/s and spontaneous nystagmus SPV < 4 °/s, all false negatives necessarily met these two conditions. This definitional result should not be interpreted as evidence that either factor has no effect on missed cases (Table 4). Detailed individual-level characteristics of the 14 false-negative cases, including bilateral warm and cold SPVs, warm and cold monothermal asymmetry values, CP and DP characteristics, spontaneous nystagmus direction and SPV, and distances from the candidate-rule thresholds, are presented in Supplementary Table S10.

**Table 4:** False negatives according to the severity of canal paresis.

CP severity	CP-positive cases	False negatives	False-negative rate (95% CI)	Exact 95% upper bound
25 to <30%	183	10	5.5% (2.7–9.8%)	9.82%
30 to <40%	224	4	1.8% (0.5–4.5%)	4.51%
≥40%	439	0	0.0% (0.0–0.8%)	0.84%

The denominator is the number of validation patients within each CP severity stratum. A zero observed false-negative count does not imply zero true risk.

### 3.6 Stratified analyses by DP and spontaneous nystagmus

Warm and cold monothermal testing both performed well at low DP. In the DP < 10% stratum, the AUC was 0.971 for warm testing and 0.965 for cold testing, and sensitivity at the 5% threshold was 100% for both. Cold performance declined more markedly with increasing DP. In the DP ≥ 30% stratum, the AUC remained 0.849 (95% CI, 0.801–0.892) for warm testing but fell to 0.369 (95% CI, 0.311–0.425) for cold testing. Specificity was 0 at the 5% threshold for both schemes in this stratum, indicating that no CP-negative patient entered the candidate-stop group at this threshold. This does not imply that the tests lacked value for all purposes. The cold AUC below 0.5 indicated marked loss of ranking ability and possible direction reversal, but did not establish a causal effect of DP (Supplementary Table S7; Figure S7).

Across spontaneous nystagmus SPV strata of 0, >0 to <2, 2 to <4, and ≥4 °/s, the warm AUCs were 0.890, 0.905, 0.873, and 0.765, respectively; the corresponding cold AUCs were 0.802, 0.765, 0.800, and 0.522. The ≥4 °/s stratum included only 95 patients, of whom 46 were CP positive and 49 were CP negative, resulting in wide confidence intervals. The 100% point estimate for warm sensitivity at the 5% threshold should therefore be interpreted together with its exact 95% CI of 92.3–100.0% (Supplementary Table S8; Figure S6).

### 3.7 Cross-temperature schemes and sensitivity analyses

The warm-to-cold standardization coefficient k was estimated as 1.287 in 706 derivation-cohort patients with low CP, low DP, and spontaneous nystagmus SPV = 0. In the validation cohort, the RW + LC scheme had an AUC of 0.833, sensitivity of 96.8%, specificity of 16.9%, and NPV of 90.2%. The LW + RC scheme had an AUC of 0.826, sensitivity of 97.3%, specificity of 15.3%, and NPV of 90.7%. Both schemes used the 5% derivation threshold. Because stimulus temperature and ear side could not be separated, neither scheme was included in the clinical recommendation (Supplementary Table S9).

When the reference threshold was changed to CP ≥ 20%, the final rule had a validation-cohort sensitivity of 95.2%, specificity of 40.6%, and NPV of 90.3%. At CP ≥ 30%, the corresponding values were 99.4, 32.8, and 99.3%; at CP ≥ 40%, they were 100.0, 29.1, and 100.0%. The lower sensitivity for the CP ≥ 20% endpoint indicated that false-negative risk was concentrated among milder abnormalities. Full results are provided in Supplementary Table S3.

### 3.8 Comparison with BSA-like criteria and a warm-MCA-only strategy

In the validation cohort, the BSA-like criteria yielded TP/FN/TN/FP counts of 827/19/554/916, sensitivity of 97.8%, specificity of 37.7%, NPV of 96.7%, and a candidate stop rate of 24.7%. Compared with the BSA-like criteria, the final candidate rule had 0.6 percentage points higher sensitivity (95% CI, −0.1 to 1.3; McNemar *p* = 0.180), 1.4 percentage points lower specificity (95% CI, −3.1 to 0.3; *p* = 0.096), and 0.8 percentage points higher NPV (95% CI, −0.2 to 1.9). Per 1,000 patients, the final rule missed 2.2 fewer cases with CP ≥ 25% (95% CI, −4.7 to 0.4) but avoided 22.5 fewer cold irrigations (Table 5; Figure 5).

**Table 5:** Comparison of the final composite rule with BSA-like and warm-MCA-only strategies in the temporal validation cohort.

Rule	MCA threshold (%)	Minimum warm SPV threshold (°/s)	Spontaneous nystagmus condition	TP/FN/TN/FP	Sensitivity (95% CI)	Specificity (95% CI)	NPV (95% CI)	Candidate stop rate (95% CI)	Missed CP ≥ 25% per 1,000	Avoided cold irrigations per 1,000
*Panel A. Direct comparison of diagnostic performance*										
Final composite rule	<17.5	>11	SPV < 4°/s	832/14/533/937	98.3% (97.2–99.1%)	36.3% (33.8–38.8%)	97.4% (95.7–98.6%)	23.6% (21.9–25.4%)	6.0	472.4
BSA-like criteria	<15	>8	SPV ≤ 4°/s	827/19/554/916	97.8% (96.5–98.6%)	37.7% (35.2–40.2%)	96.7% (94.9–98.0%)	24.7% (23.0–26.6%)	8.2	494.8
Warm-MCA-only rule	<5%	None	None	837/9/229/1241	98.9%	15.6%	96.2%	10.3%	—	—
The numerical thresholds for the BSA-like comparator were taken from Appendix C of the 2010 British Society of Audiology recommended procedure. This post hoc comparison was not used for selection or retuning of the final rule.										

Comparison	Difference (final−BSA-like), 95% CI	McNemar *p*-value	Final stop/BSA complete	Final complete/BSA stop
*Panel B. Paired comparison between the final composite rule and BSA-like criteria*				
Sensitivity	0.6 pp. (−0.1 to 1.3)	0.180	2	7
Specificity	−1.4 pp. (−3.1 to 0.3)	0.096	62	83
Negative predictive value	0.8 pp. (−0.2 to 1.9)	—	—	—
Candidate stop rate	−1.1 pp. (−2.2 to −0.1)	—	—	—
Missed CP ≥ 25% cases per 1,000	−2.2 (−4.7 to 0.4)	—	—	—
Differences are expressed as final composite rule minus BSA-like criteria. For sensitivity, discordant counts were calculated among patients with CP ≥25%; for specificity, they were calculated among patients with CP <25%. “Stop” denotes meeting the candidate-stop criteria, whereas “complete” denotes requiring complete bithermal testing. McNemar p values apply to paired sensitivity and specificity comparisons; other differences use patient-level bootstrap 95% confidence intervals.				

Metric	Final−MCA only	95% CI	*p*-value
*Panel C. Paired comparison between the final composite rule and the derivation-selected warm-MCA-only rule*			
Sensitivity	−0.6 pp	−1.6 to 0.4	0.383
Specificity	+20.7 pp	18.1 to 23.4	<0.001
NPV	+1.2 pp	−1.3 to 3.8	—
Candidate-stop rate	+13.3 pp	11.6 to 15.2	<0.001
Missed CP ≥ 25% per 1,000	+2.2	−1.3 to 6.0	—

**Figure 5:**
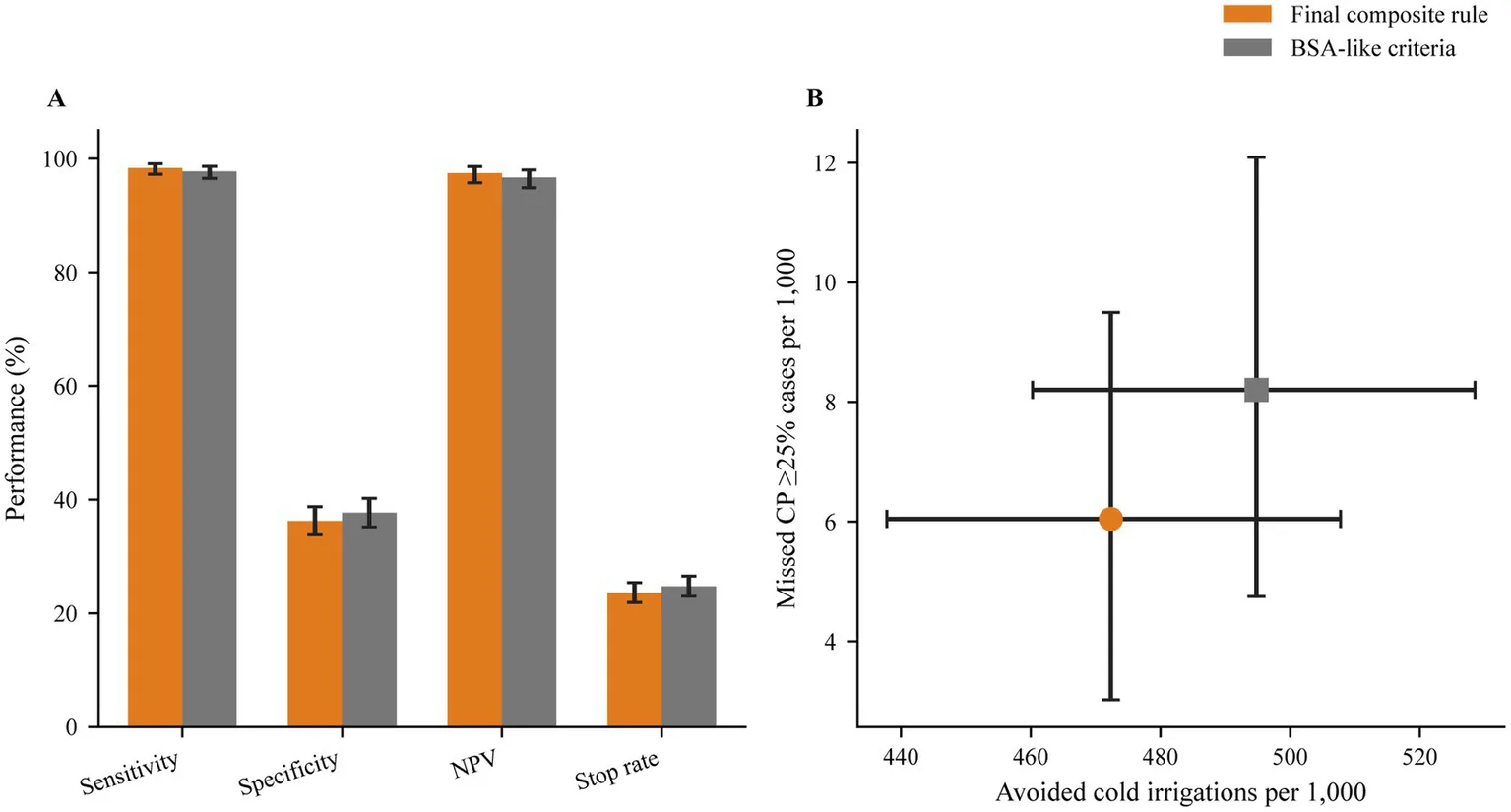
Local comparison of the final composite rule and BSA-like criteria in the same temporal validation cohort (*n* = 2,316). **A** Diagnostic performance with exact 95% confidence intervals. **B** Safety–efficiency trade-off with patient-level bootstrap 95% confidence intervals (2,000 resamples). The BSA-like criteria were defined as absolute warm MCA < 15%, both warm responses >8°/s, and spontaneous nystagmus SPV ≤ 4°/s, following Appendix C of the 2010 British Society of Audiology recommended procedure. Differences were evaluated with paired methods and should not be interpreted as proof of superiority.

Under the same derivation-cohort sensitivity constraint of ≥97.5%, the selected warm-MCA-only rule used a 5% threshold. In the validation cohort, this rule yielded TP/FN/TN/FP counts of 837/9/229/1,241, corresponding to a sensitivity of 98.9% (95% CI, 98.0–99.5%), specificity of 15.6% (13.8–17.5%), NPV of 96.2% (92.9–98.3%), and candidate-stop rate of 10.3% (9.1–11.6%).

Compared with the warm-MCA-only rule, the final composite rule had 0.6 percentage points lower sensitivity (95% CI, −1.6 to 0.4; McNemar *p* = 0.383), 20.7 percentage points higher specificity (18.1–23.4; *p* < 0.001), and a 13.3-percentage-point higher candidate-stop rate (11.6–15.2; *p* < 0.001). The NPV difference was 1.2 percentage points (−1.3 to 3.8). The composite rule produced 14 false negatives compared with 9 for the MCA-only rule, corresponding to a difference of 2.2 missed CP ≥ 25% cases per 1,000 patients (−1.3 to 6.0). Thus, the additional criteria increased specificity and candidate-stop yield under the common derivation sensitivity constraint, but did not reduce the observed number of false negatives.

None of the paired differences in sensitivity, specificity, NPV, or missed CP ≥ 25% cases demonstrated statistically significant superiority of the final rule over the BSA-like criteria. The final rule also had a slightly lower candidate-stop rate than the BSA-like comparator (23.6% vs. 24.7%). Accordingly, the present data do not establish a superior false-negative–irrigation-reduction balance for the newly derived rule.

## 4 Discussion

In this large air-caloric cohort tested using a fixed warm-before-cold sequence, bilateral warm MCA showed substantially better discrimination for abnormal CP on complete bithermal testing than bilateral cold MCA, with AUCs of 0.888 and 0.777, respectively. After warm MCA was combined with minimum warm response strength and spontaneous nystagmus SPV, the final candidate rule achieved 98.3% sensitivity, 36.3% specificity, and a 97.4% NPV in the temporal validation cohort, while 23.6% of patients met the candidate-stop criteria. These findings support warm monothermal testing as a triage step for a subset of low-risk patients rather than as a replacement for complete bithermal testing. The higher observed discrimination of warm MCA within this fixed-sequence protocol was broadly consistent with reports by Murnane et al. (11), Lightfoot et al. (10), Thatcher et al. (12), and Alhabib and Saliba (5). In 1,002 patients, Murnane et al. (11) found that warm monothermal testing outperformed cold testing under three of four bithermal reference standards. A 10% warm interaural difference produced an approximately 1% false-negative rate when unilateral weakness (UW) ≥ 25% defined abnormality. Alhabib and Saliba likewise reported a higher AUC for warm than cold MCA (0.855 vs. 0.759). Our estimates were similar and extend this evidence to a substantially larger cohort tested with air irrigation. However, because both warm irrigations always preceded both cold irrigations, stimulus temperature was completely confounded with test order. The 5-min inter-irrigation interval and the requirement for resolution of the preceding response reduced, but could not eliminate, possible adaptation, fatigue, residual-response, or other order effects. Therefore, the present findings support better discrimination by warm MCA within the specific warm-before-cold protocol evaluated here, rather than establishing an intrinsic superiority of warm stimulation independently of test order.

Not all previous studies have favored warm monothermal testing. Enticott et al. (28) used stringent criteria of MCA < 5% and no spontaneous nystagmus in 744 examinations and reported a false-negative rate below 1% for cold testing, compared with below 7.1% for warm testing. Cunha et al. (29) also found slightly higher sensitivity and specificity for 30 °C cold water than for 44 °C warm water, although the differences were not statistically significant. However, the stringent criteria used by Enticott et al. (28) required more than three-quarters of patients with normal bithermal results to complete the full test. The very low false-negative rate was therefore achieved at the cost of limited screening efficiency.

Differences between warm and cold monothermal testing may partly reflect the interaction between CP and DP. Complete bithermal CP combines warm and cold responses by ear, whereas DP combines responses by nystagmus direction. DP may therefore amplify interaural asymmetry at one temperature while attenuating it at the other. In our data, both warm and cold testing performed well at low DP, whereas cold performance declined more markedly as DP increased. In the DP ≥ 30% validation subgroup, the warm AUC remained 0.849 while the cold AUC fell to 0.369. High DP may therefore help explain failure of cold monothermal screening, although the observational data do not establish causality. Variation in DP distribution, disease mix, and the prevalence of spontaneous nystagmus across studies may also contribute to inconsistent conclusions about warm and cold testing. Overall sensitivity at one fixed temperature should not be assumed to generalize to every laboratory population.

Early studies recognized that interaural asymmetry alone was insufficient to exclude abnormal bithermal results with high sensitivity. Barber et al. (9) recommended considering both interaural difference and minimum SPV, and Jacobson and Means (30) additionally considered spontaneous or positional nystagmus and oculomotor findings. Jacobson et al. (31) subsequently showed in 504 patients that incorporating other electronystagmographic failure criteria could further improve warm monothermal screening. These findings support our decision to combine MCA, WarmMin, and spontaneous nystagmus. Based on Lightfoot et al. (10), the BSA recommends considering termination of subsequent cold irrigations when warm MCA is <15%, both warm responses are >8 °/s, and mean or peak spontaneous nystagmus SPV is ≤4 °/s. That combination was reported to have approximately 95% sensitivity and 71% specificity. Our final rule used MCA < 17.5%, WarmMin >11 °/s, and spontaneous nystagmus SPV < 4 °/s. Compared with the BSA criteria, it allowed slightly greater interaural asymmetry but imposed a higher minimum response threshold and a marginally stricter spontaneous nystagmus boundary. In the direct paired comparison within the present validation cohort, the final composite rule and the BSA-like criteria did not differ significantly in sensitivity, specificity, NPV, or missed CP ≥ 25% cases. The final rule also produced a slightly lower candidate-stop rate. Comparisons with sensitivity and specificity estimates reported in previous BSA-related studies involve different populations, protocols, and reference definitions and should not be interpreted as evidence of superiority. The present data therefore do not demonstrate that the newly derived thresholds provide a superior balance between false-negative frequency and potential irrigation reduction. Compared with the warm-MCA-only rule selected under the same derivation sensitivity constraint, the composite rule substantially increased specificity and candidate-stop yield without a statistically significant change in sensitivity. However, it did not reduce the observed false-negative count. These findings support incremental value primarily in identifying more CP-negative patients who could potentially stop, rather than in improving sensitivity or clinical safety. The comparison evaluates the composite strategy as a whole and does not isolate the independent contribution of WarmMin and spontaneous nystagmus. The Lightfoot criteria are closer to a balanced sensitivity-specificity operating point. The preferred rule therefore depends on the false-negative risk acceptable to a laboratory and the desired reduction in irrigations.

In a prospective cohort of 366 patients, Thatcher et al. (12) used a 25% warm monothermal interaural difference threshold and reported 98.0% sensitivity, 91.3% specificity, a negative screening result in 71.9% of patients, and an average reduction in test time of approximately 15 min. This efficiency was substantially greater than the 23.6% candidate stop rate in our study, but the two studies are not directly comparable. Thatcher et al. (12) used water irrigation and excluded patients who were ineligible or failed screening, whereas our analysis was restricted to patients with complete four-irrigation air caloric data. Incomplete examinations, including some terminated because of intolerance, were therefore excluded. This may have selectively excluded patients who might benefit most from a shorter protocol. Our composite rule additionally incorporated spontaneous nystagmus and minimum SPV. Differences in reference standard, case mix, abnormality prevalence, and failure criteria can materially affect specificity and NPV. Moreover, the 71.9% negative screening rate reported by Thatcher et al. (12) and our 23.6% rate of meeting all candidate-stop criteria do not represent identical denominators or decision rules. Murnane et al. (11) reported that a warm interaural difference of 10% could reduce complete testing in approximately 40% of patients without UW. That estimate used patients without UW as the denominator, whereas our 23.6% used the entire validation cohort. Presenting both the theoretically avoided irrigations and CP-positive cases that would have been missed per 1,000 patients provides a direct description of the retrospective trade-off between irrigation reduction and classification error.

Spontaneous nystagmus has long been a contentious factor in monothermal screening. The BSA permits patients with a mean or peak spontaneous nystagmus SPV ≤ 4 °/s to meet its stopping criteria, while noting that the effects of spontaneous nystagmus on warm and cold responses are incompletely understood and that simple arithmetic correction should not be applied mechanically (24). Several approaches have been proposed for patients with spontaneous nystagmus. In a retrospective analysis of 2,304 patients, Domínguez-Durán et al. (32) included the spontaneous nystagmus value in the Jongkees formula and used a two-threshold gray-zone strategy. This allowed some patients with spontaneous nystagmus or nystagmus reversal to undergo monothermal screening and reduced bithermal stimulations by approximately 38%, with sensitivity and specificity of about 95%. The finding indicates that spontaneous nystagmus need not always be an absolute exclusion criterion, but it must be considered when calculating and interpreting monothermal asymmetry. Simple mathematical correction may nevertheless be insufficient. Among 259 patients studied by Alhabib and Saliba, 34 had horizontal spontaneous nystagmus with a mean SPV of 2.25 °/s. Correction for spontaneous nystagmus increased warm-test sensitivity from 61.5 to 81.0%, but reduced specificity from 75.0 to 53.8%, and NPV remained only 63.6% (5). Thus, improved sensitivity may be offset by more false positives and lower specificity. Directional effects of spontaneous nystagmus may also operate through DP. In 78 patients with acute vertigo and unidirectional horizontal spontaneous nystagmus, Gufoni et al. (33) found that DP direction usually agreed with spontaneous nystagmus direction. Using the algebraic relation among CP, DP, and bilateral cold responses, they proposed that cold monothermal responses could assist in identifying pathological unilateral weakness in selected patients. This work illustrates that interaural differences at one temperature may reflect both true labyrinthine asymmetry and a directional nystagmus offset. Interpretation may therefore require the direction of spontaneous nystagmus, CP, and DP. DP itself, however, is not equivalent to unilateral peripheral vestibular hypofunction. Halmagyi et al. (34) screened 15,542 bithermal tests and identified patients with isolated DP ≥ 40%, spontaneous nystagmus ≤2 °/s, and CP ≤ 25%; 114 patients were ultimately analyzed. Isolated DP occurred in benign paroxysmal positional vertigo, Ménière disease, migraine-associated vertigo, and some central disorders, although many patients had no definitive diagnosis. Isolated DP therefore lacks specific localizing value and should not independently determine lesion side or site. In the present study, spontaneous nystagmus was not treated as an automatic exclusion. The VNG system applied direction-dependent baseline correction to each of the four caloric responses, while the absolute spontaneous-nystagmus SPV was additionally evaluated as a separate component of the composite candidate rule. Multiple spontaneous-nystagmus SPV limits were evaluated jointly with MCA and WarmMin. Relaxing only the spontaneous nystagmus threshold while holding MCA and WarmMin fixed reduced sensitivity. Joint optimization of all three parameters was required to improve the candidate stop rate while maintaining high sensitivity. Spontaneous nystagmus should therefore be considered as one component of a composite rule, whereas DP is more appropriately used as a stratification and explanatory variable than as a reference standard for unilateral weakness.

Diagnostic uncertainty in monothermal screening was concentrated among mild unilateral weakness near the normal-abnormal boundary. A systematic review by Adams et al. (13) included 15 studies and 5,572 participants. Although monothermal and bithermal asymmetry measures were correlated overall, the association was unstable in subgroups with mild UW, and studies excluding severe unilateral weakness generally reported lower sensitivity. Keith et al. (35) found stable correlations when UW was <15% or >30%, but no significant association between warm monothermal and bithermal results when UW was 15–30%. Murnane et al. (11) also showed that lowering the abnormality threshold from UW ≥ 25% to UW ≥ 20% increased false negatives by including more mildly abnormal cases. Our findings were consistent with this pattern. The final rule produced 14 false negatives with CP ≥ 25% in the temporal validation cohort: 10 had CP from 25 to <30%, four had CP from 30 to <40%, and none had CP ≥ 40%. Missed cases were therefore concentrated among borderline and mild-to-moderate abnormalities, rather than clearly severe CP. The absence of observed false negatives with CP ≥ 40% does not imply zero true risk and requires confirmation in an independent external sample. Monothermal studies should therefore report false negatives by CP severity rather than sensitivity alone. Domínguez-Durán et al. (32) used spontaneous nystagmus correction, a two-threshold gray zone, and a maximum-utility threshold in 2,304 patients. Their strategy reduced bithermal stimulation by approximately 38% while maintaining sensitivity and specificity near 95%. Our present approach used a binary rule: patients meeting all criteria were candidate stops and all others continued bithermal testing. Future work could evaluate a three-zone strategy comprising a low-risk stop zone, an intermediate gray zone, and a definite-completion zone. Patients with MCA, spontaneous nystagmus SPV, or WarmMin close to the decision boundary could enter the gray zone. Such a strategy might improve workflow flexibility while avoiding the interpretation of data-derived thresholds as absolute biological boundaries.

Most monothermal screening studies have used bilateral stimulation at the same temperature, and evidence for pairing warm stimulation of one ear with cold stimulation of the contralateral ear is limited. Norré retrospectively reviewed 272 complete bithermal tests and found substantial false-positive and false-negative rates with either bilateral warm or bilateral cold stimulation. An alternating bithermal approach was proposed to shorten testing, but the abstract did not report the specific stimulus pairs, calculation, or decision threshold and therefore does not directly validate our cross-temperature schemes (36). Physiologically, right warm and left cold stimulation both induce right-beating nystagmus, whereas left warm and right cold stimulation both induce left-beating nystagmus. RW + LC and LW + RC therefore resemble the two directional components of complete bithermal testing more than a direct bilateral comparison. These schemes completely confound ear side, stimulus temperature, and nystagmus direction. They are also susceptible to spontaneous nystagmus and DP, making it difficult to distinguish true unilateral labyrinthine asymmetry from a directional nystagmus offset using only two irrigations. The relation of cold responses to CP and DP in the presence of spontaneous nystagmus also depends on nystagmus direction (33). Differences in response strength and stimulus order may further affect cross-temperature schemes. Furman and Jacob found that caloric responses could decrease across sequential stimulations and produce systematic bias in unilateral weakness under some orders (19). Noaksson et al. (37) reported that warm-first versus cold-first testing altered the response-strength difference between warm and cold stimulation, although effects on complete Jongkees indices were small. With only two irrigations, cross-temperature schemes lack the balancing provided by all four responses and may be more vulnerable to temperature and order effects. In our data, the cross-temperature AUCs were approximately 0.83 and sensitivity approached 97%, but NPV and specificity were both lower than for the final bilateral warm composite rule. These schemes should therefore remain exploratory and should not enter clinical recommendations without prospective validation of the standardization method, stimulus order, and diagnostic performance in a prespecified independent cohort.

This study has several strengths. First, the sample of 7,753 patients was larger than those of most previous individual studies and supported separate derivation and temporal validation cohorts. Second, the 50 °C/24 °C air protocol matched the air temperatures recommended by the BSA and was directly relevant to routine laboratory practice. The BSA nevertheless emphasizes that air caloric effectiveness depends on equipment and nozzle characteristics and that each laboratory should establish its own normative values. Third, within a uniform fixed-sequence clinical protocol, we directly compared bilateral warm and bilateral cold testing and systematically examined the influence of DP and spontaneous nystagmus. Fourth, rule selection prioritized control of false negatives before optimizing triage efficiency, rather than relying solely on the Youden index. This approach better matched the clinical purpose of stopping after a negative screen. Finally, reporting missed cases and the theoretical reduction in irrigations per 1,000 patients, together with the severity distribution of false negatives, made the trade-off between irrigation reduction and CP false-negative frequency more transparent.

### 4.1 Limitations

This study has several limitations. First, this single-center retrospective study may reflect center-specific case mix, air-irrigation equipment, and laboratory procedures. Inclusion required complete quantitative data from all four irrigations. Some examinations were terminated because of physical symptoms, intolerance, anxiety, or refusal, so complete-case selection may have underrepresented patients most likely to benefit from shorter testing. True patient identifiers were unavailable, and repeat examinations were identified using normalized name and sex. Records sharing the same candidate identifier were assigned to only one cohort, but rare linkage errors cannot be excluded. Second, all examinations followed the fixed sequence of right warm, left warm, right cold, and left cold irrigations. Stimulus temperature was therefore completely confounded with test order. Although irrigations were separated by 5 min and testing resumed only after nystagmus and vertigo had resolved, adaptation, fatigue, residual responses, or other order effects remain possible. The greater discrimination of warm testing therefore applies to the evaluated warm-first protocol and cannot be attributed exclusively to stimulus temperature. Third, the temporal validation cohort was not completely untouched during rule development. The final 200-rule ranking and numerical threshold selection used the derivation cohort alone, and validation metrics were not included in the formal ranking algorithm. However, validation-cohort AUCs, performance at individual MCA thresholds, and results from earlier candidate rules had been reviewed before all elements of the analytical framework were finalized. The framework was not prospectively registered, and some elements of the selection hierarchy were finalized during refinement of the derivation-stage analysis. This prior access may have introduced optimistic bias. Moreover, the 97.5% sensitivity constraint was applied to the point estimate rather than its lower confidence limit, and no maximum acceptable number of false negatives was prespecified. Meeting this empirical constraint therefore does not establish a true sensitivity of at least 97.5%. The proportion with spontaneous nystagmus SPV = 0 was lower in the validation cohort than in the derivation cohort (38.0% vs. 48.2%). Although the proportions meeting the final SPV < 4 °/s criterion were similar, this shift may limit temporal transportability. The findings should therefore be regarded as internal temporal validation and require confirmation in a prospective cohort with the analysis plan and thresholds locked in advance. Fourth, monothermal MCA and the bithermal CP reference standard were calculated from overlapping caloric responses and were mathematically coupled. The reported performance therefore represents prediction of dichotomized bithermal CP rather than validation against an independent measure of vestibular dysfunction, and temporal separation does not remove this dependence. The rule was developed specifically for CP ≥ 25% and should not be applied in laboratories using CP ≥ 20% without separate validation. The NPV of 97.4% was observed at a CP ≥ 25% prevalence of 36.5% (846/2,316); PPV and NPV may differ in populations with different referral patterns or CP prevalences. Fifth, complete clinical diagnoses, actual examination times, and adverse-event data were unavailable. Reductions in irrigation count are retrospective theoretical estimates and cannot be interpreted as observed improvements in examination time, tolerance, nausea, vomiting, or completion. The rule was not used prospectively to stop testing. Safety therefore refers only to the observed false-negative frequency relative to bithermal CP classification, not to clinical safety, diagnostic management, patient outcomes, or adverse events. Caloric hyperresponsiveness was not evaluated, so meeting the candidate-stop criteria does not exclude abnormally high responses. Finally, 14 false negatives with CP ≥ 25% remained in the validation cohort. The absence of observed false negatives among patients with CP ≥ 40% does not establish zero risk of missing severe abnormalities. The exploratory cross-temperature schemes also remained confounded by temperature and ear side. All candidate rules require independent prospective validation before clinical implementation.

## 5 Conclusion

Within the fixed warm-before-cold air-irrigation protocol evaluated in this study, bilateral warm monothermal testing showed better overall discrimination than bilateral cold monothermal testing. The final derivation-cohort rule required |MCA_W| < 17.5%, WarmMin >11 °/s, and spontaneous nystagmus SPV < 4 °/s. In the internal temporal validation cohort, the rule identified 23.6% of patients as candidate stops, with 98.3% sensitivity and a 97.4% NPV, but still produced 14 false negatives with CP ≥ 25%. These retrospective findings support the rule as a candidate for prospective triage evaluation but do not establish clinical safety or observed improvements in examination time, tolerability, completion, or adverse events. The rule should not replace complete bithermal testing and requires validation in an independent prospective cohort.

## Data Availability

The original contributions presented in the study are included in the article/Supplementary material, further inquiries can be directed to the corresponding authors.
