# Supplementary material for: Diagnostic accuracy of warm and cold monothermal air caloric screening for bithermal canal paresis and derivation of a warm-test triage rule: a retrospective temporal validation study
Source: Front Neurol. 2026 Oct 14;17:1953594. doi: 10.3389/fneur.2026.1953594 (PMC13713255; doi:10.3389/fneur.2026.1953594)
Supplement: Supplementary file 1 [file Supplementary_file_1.docx]

**Supplementary Material**

**Supplementary Figures**

Supplementary Figure S1. Distributions of peak slow-phase velocity across the four caloric stimulations in the main analysis cohort. Histograms show peak slow-phase velocity (SPV) following right warm 50°C (RW), left warm 50°C (LW), right cold 24°C (RC), and left cold 24°C (LC) air irrigations (n = 7,753). Warm irrigations are shown in orange and cold irrigations in blue. All panels use a bin width of 5°/s and display the full observed range. The x-axis represents SPV (°/s), and the y-axis represents the number of patients.

Supplementary Figure S2. Association between absolute warm monothermal caloric asymmetry (MCA) and absolute canal paresis in the temporal validation cohort (n = 2,316). The horizontal dashed line denotes CP = 25% and the vertical dotted line denotes the 17.5% MCA component of the final warm composite rule. The upper-left region represents potential false negatives for the MCA component alone. The solid line connects binned medians and is descriptive.

Supplementary Figure S3. Association between absolute cold monothermal caloric asymmetry (MCA) and absolute canal paresis in the temporal validation cohort (n = 2,316). The horizontal dashed line denotes CP = 25% and the vertical dotted line denotes the prespecified 5% single-cold-MCA benchmark. The upper-left region represents potential false negatives for the MCA component alone. The solid line connects binned medians and is descriptive.

Supplementary Figure S4. Effect of varying the minimum warm SPV threshold while holding the other two components at the final selected values (absolute warm MCA <17.5% and spontaneous nystagmus SPV <4°/s) in the derivation cohort. a Sensitivity. b Candidate stop rate. The vertical dotted line denotes the selected minimum warm SPV threshold; the horizontal dashed line denotes the prespecified 97.5% sensitivity criterion.

Supplementary Figure S5. Sensitivity, specificity, negative predictive value, and candidate stop rate of the final warm composite rule in the derivation (n = 5,437) and temporal validation (n = 2,316) cohorts. Error bars are Clopper–Pearson exact 95% confidence intervals. The y-axis is discontinuous, omitting the 45%–90% interval, which contained no point estimates or confidence intervals.

Supplementary Figure S6. Warm and cold monothermal performance across unified spontaneous nystagmus SPV strata in the temporal validation cohort. a AUC with 2,000-sample stratified bootstrap 95% confidence intervals. b Sensitivity at the prespecified 5% single-MCA benchmark with Clopper–Pearson exact 95% confidence intervals. Numbers of CP-positive and CP-negative patients in each stratum are reported in the corresponding table. The y-axis ranges were truncated to improve visualization; all point estimates and 95% confidence intervals are displayed in full.

Supplementary Figure S7. Warm and cold monothermal performance across absolute directional preponderance strata in the temporal validation cohort. a AUC with 2,000-sample stratified bootstrap 95% confidence intervals. b Sensitivity at the prespecified 5% single-MCA benchmark with Clopper–Pearson exact 95% confidence intervals. Numbers of CP-positive and CP-negative patients in each stratum are reported in the corresponding table. The y-axis ranges were truncated to improve visualization; all point estimates and 95% confidence intervals are displayed in full.

**Supplementary Tables**

Supplementary Table S1. Performance across individual MCA thresholds

| **Cohort** | **Scheme** | **MCA threshold (%)** | **TP** | **FN** | **TN** | **FP** | **Sensitivity (95% CI)** | **Specificity (95% CI)** | **NPV (95% CI)** | **LR− (95% CI)** | **Candidate stop rate (95% CI)** |
| --- | --- | --- | --- | --- | --- | --- | --- | --- | --- | --- | --- |
| Derivation cohort | Warm | 5 | 1,916 | 36 | 622 | 2,863 | 98.2% (97.5%–98.7%) | 17.8% (16.6%–19.2%) | 94.5% (92.5%–96.1%) | 0.103 (0.074–0.144) | 12.1% (11.2%–13.0%) |
| Derivation cohort | Warm | 7.5 | 1,902 | 50 | 888 | 2,597 | 97.4% (96.6%–98.1%) | 25.5% (24.0%–27.0%) | 94.7% (93.0%–96.0%) | 0.101 (0.076–0.133) | 17.3% (16.3%–18.3%) |
| Derivation cohort | Warm | 10 | 1,882 | 70 | 1,150 | 2,335 | 96.4% (95.5%–97.2%) | 33.0% (31.4%–34.6%) | 94.3% (92.8%–95.5%) | 0.109 (0.086–0.137) | 22.4% (21.3%–23.6%) |
| Derivation cohort | Warm | 12.5 | 1,864 | 88 | 1,404 | 2,081 | 95.5% (94.5%–96.4%) | 40.3% (38.7%–41.9%) | 94.1% (92.8%–95.2%) | 0.112 (0.091–0.138) | 27.4% (26.3%–28.6%) |
| Derivation cohort | Warm | 15 | 1,835 | 117 | 1,667 | 1,818 | 94.0% (92.9%–95.0%) | 47.8% (46.2%–49.5%) | 93.4% (92.2%–94.5%) | 0.125 (0.105–0.150) | 32.8% (31.6%–34.1%) |
| Derivation cohort | Warm | 17.5 | 1,811 | 141 | 1,871 | 1,614 | 92.8% (91.5%–93.9%) | 53.7% (52.0%–55.4%) | 93.0% (91.8%–94.1%) | 0.135 (0.114–0.158) | 37.0% (35.7%–38.3%) |
| Derivation cohort | Warm | 20 | 1,786 | 166 | 2,059 | 1,426 | 91.5% (90.2%–92.7%) | 59.1% (57.4%–60.7%) | 92.5% (91.4%–93.6%) | 0.144 (0.124–0.167) | 40.9% (39.6%–42.2%) |
| Derivation cohort | Warm | 22.5 | 1,718 | 234 | 2,335 | 1,150 | 88.0% (86.5%–89.4%) | 67.0% (65.4%–68.6%) | 90.9% (89.7%–92.0%) | 0.179 (0.158–0.202) | 47.3% (45.9%–48.6%) |
| Derivation cohort | Warm | 25 | 1,680 | 272 | 2,477 | 1,008 | 86.1% (84.4%–87.6%) | 71.1% (69.5%–72.6%) | 90.1% (88.9%–91.2%) | 0.196 (0.175–0.219) | 50.6% (49.2%–51.9%) |
| Derivation cohort | Warm | 30 | 1,547 | 405 | 2,804 | 681 | 79.3% (77.4%–81.0%) | 80.5% (79.1%–81.8%) | 87.4% (86.2%–88.5%) | 0.258 (0.236–0.282) | 59.0% (57.7%–60.3%) |
| Derivation cohort | Cold | 5 | 1,858 | 94 | 644 | 2,841 | 95.2% (94.1%–96.1%) | 18.5% (17.2%–19.8%) | 87.3% (84.6%–89.6%) | 0.261 (0.211–0.321) | 13.6% (12.7%–14.5%) |
| Derivation cohort | Cold | 7.5 | 1,808 | 144 | 963 | 2,522 | 92.6% (91.4%–93.7%) | 27.6% (26.2%–29.2%) | 87.0% (84.9%–88.9%) | 0.267 (0.226–0.315) | 20.4% (19.3%–21.5%) |
| Derivation cohort | Cold | 10 | 1,754 | 198 | 1,264 | 2,221 | 89.9% (88.4%–91.2%) | 36.3% (34.7%–37.9%) | 86.5% (84.6%–88.2%) | 0.280 (0.243–0.321) | 26.9% (25.7%–28.1%) |
| Derivation cohort | Cold | 12.5 | 1,702 | 250 | 1,553 | 1,932 | 87.2% (85.6%–88.6%) | 44.6% (42.9%–46.2%) | 86.1% (84.5%–87.7%) | 0.287 (0.255–0.325) | 33.2% (31.9%–34.4%) |
| Derivation cohort | Cold | 15 | 1,625 | 327 | 1,835 | 1,650 | 83.2% (81.5%–84.9%) | 52.7% (51.0%–54.3%) | 84.9% (83.3%–86.4%) | 0.318 (0.287–0.353) | 39.8% (38.5%–41.1%) |
| Derivation cohort | Cold | 17.5 | 1,562 | 390 | 2,046 | 1,439 | 80.0% (78.2%–81.8%) | 58.7% (57.1%–60.3%) | 84.0% (82.5%–85.4%) | 0.340 (0.310–0.374) | 44.8% (43.5%–46.1%) |
| Derivation cohort | Cold | 20 | 1,525 | 427 | 2,178 | 1,307 | 78.1% (76.2%–79.9%) | 62.5% (60.9%–64.1%) | 83.6% (82.1%–85.0%) | 0.350 (0.321–0.382) | 47.9% (46.6%–49.3%) |
| Derivation cohort | Cold | 22.5 | 1,421 | 531 | 2,459 | 1,026 | 72.8% (70.8%–74.8%) | 70.6% (69.0%–72.1%) | 82.2% (80.8%–83.6%) | 0.386 (0.357–0.416) | 55.0% (53.7%–56.3%) |
| Derivation cohort | Cold | 25 | 1,363 | 589 | 2,586 | 899 | 69.8% (67.7%–71.9%) | 74.2% (72.7%–75.6%) | 81.4% (80.1%–82.8%) | 0.407 (0.379–0.436) | 58.4% (57.1%–59.7%) |
| Derivation cohort | Cold | 30 | 1,188 | 764 | 2,891 | 594 | 60.9% (58.7%–63.0%) | 83.0% (81.7%–84.2%) | 79.1% (77.7%–80.4%) | 0.472 (0.446–0.500) | 67.2% (66.0%–68.5%) |
| Temporal validation cohort | Warm | 5 | 837 | 9 | 229 | 1,241 | 98.9% (98.0%–99.5%) | 15.6% (13.8%–17.5%) | 96.2% (92.9%–98.3%) | 0.068 (0.035–0.132) | 10.3% (9.1%–11.6%) |
| Temporal validation cohort | Warm | 7.5 | 830 | 16 | 354 | 1,116 | 98.1% (96.9%–98.9%) | 24.1% (21.9%–26.4%) | 95.7% (93.1%–97.5%) | 0.079 (0.048–0.129) | 16.0% (14.5%–17.5%) |
| Temporal validation cohort | Warm | 10 | 818 | 28 | 448 | 1,022 | 96.7% (95.3%–97.8%) | 30.5% (28.1%–32.9%) | 94.1% (91.6%–96.1%) | 0.109 (0.075–0.158) | 20.6% (18.9%–22.3%) |
| Temporal validation cohort | Warm | 12.5 | 810 | 36 | 541 | 929 | 95.7% (94.2%–97.0%) | 36.8% (34.3%–39.3%) | 93.8% (91.5%–95.6%) | 0.116 (0.083–0.160) | 24.9% (23.2%–26.7%) |
| Temporal validation cohort | Warm | 15 | 801 | 45 | 658 | 812 | 94.7% (92.9%–96.1%) | 44.8% (42.2%–47.3%) | 93.6% (91.5%–95.3%) | 0.119 (0.089–0.159) | 30.4% (28.5%–32.3%) |
| Temporal validation cohort | Warm | 17.5 | 796 | 50 | 748 | 722 | 94.1% (92.3%–95.6%) | 50.9% (48.3%–53.5%) | 93.7% (91.8%–95.3%) | 0.116 (0.088–0.153) | 34.5% (32.5%–36.4%) |
| Temporal validation cohort | Warm | 20 | 788 | 58 | 811 | 659 | 93.1% (91.2%–94.8%) | 55.2% (52.6%–57.7%) | 93.3% (91.5%–94.9%) | 0.124 (0.097–0.160) | 37.5% (35.5%–39.5%) |
| Temporal validation cohort | Warm | 22.5 | 769 | 77 | 951 | 519 | 90.9% (88.8%–92.8%) | 64.7% (62.2%–67.1%) | 92.5% (90.7%–94.0%) | 0.141 (0.113–0.175) | 44.4% (42.4%–46.4%) |
| Temporal validation cohort | Warm | 25 | 758 | 88 | 1,023 | 447 | 89.6% (87.3%–91.6%) | 69.6% (67.2%–71.9%) | 92.1% (90.3%–93.6%) | 0.149 (0.122–0.183) | 48.0% (45.9%–50.0%) |
| Temporal validation cohort | Warm | 30 | 696 | 150 | 1,177 | 293 | 82.3% (79.5%–84.8%) | 80.1% (77.9%–82.1%) | 88.7% (86.9%–90.4%) | 0.221 (0.191–0.257) | 57.3% (55.3%–59.3%) |
| Temporal validation cohort | Cold | 5 | 801 | 45 | 268 | 1,202 | 94.7% (92.9%–96.1%) | 18.2% (16.3%–20.3%) | 85.6% (81.2%–89.3%) | 0.292 (0.215–0.395) | 13.5% (12.1%–15.0%) |
| Temporal validation cohort | Cold | 7.5 | 782 | 64 | 411 | 1,059 | 92.4% (90.4%–94.1%) | 28.0% (25.7%–30.3%) | 86.5% (83.1%–89.5%) | 0.271 (0.211–0.347) | 20.5% (18.9%–22.2%) |
| Temporal validation cohort | Cold | 10 | 760 | 86 | 532 | 938 | 89.8% (87.6%–91.8%) | 36.2% (33.7%–38.7%) | 86.1% (83.1%–88.7%) | 0.281 (0.227–0.347) | 26.7% (24.9%–28.5%) |
| Temporal validation cohort | Cold | 12.5 | 741 | 105 | 652 | 818 | 87.6% (85.2%–89.7%) | 44.4% (41.8%–46.9%) | 86.1% (83.5%–88.5%) | 0.280 (0.232–0.338) | 32.7% (30.8%–34.6%) |
| Temporal validation cohort | Cold | 15 | 709 | 137 | 774 | 696 | 83.8% (81.1%–86.2%) | 52.7% (50.1%–55.2%) | 85.0% (82.5%–87.2%) | 0.308 (0.262–0.361) | 39.3% (37.3%–41.4%) |
| Temporal validation cohort | Cold | 17.5 | 680 | 166 | 859 | 611 | 80.4% (77.5%–83.0%) | 58.4% (55.9%–61.0%) | 83.8% (81.4%–86.0%) | 0.336 (0.291–0.387) | 44.3% (42.2%–46.3%) |
| Temporal validation cohort | Cold | 20 | 658 | 188 | 918 | 552 | 77.8% (74.8%–80.5%) | 62.4% (59.9%–64.9%) | 83.0% (80.7%–85.2%) | 0.356 (0.312–0.406) | 47.8% (45.7%–49.8%) |
| Temporal validation cohort | Cold | 22.5 | 608 | 238 | 1,014 | 456 | 71.9% (68.7%–74.9%) | 69.0% (66.5%–71.3%) | 81.0% (78.7%–83.1%) | 0.408 (0.364–0.457) | 54.1% (52.0%–56.1%) |
| Temporal validation cohort | Cold | 25 | 582 | 264 | 1,078 | 392 | 68.8% (65.6%–71.9%) | 73.3% (71.0%–75.6%) | 80.3% (78.1%–82.4%) | 0.426 (0.383–0.472) | 57.9% (55.9%–60.0%) |
| Temporal validation cohort | Cold | 30 | 510 | 336 | 1,205 | 265 | 60.3% (56.9%–63.6%) | 82.0% (79.9%–83.9%) | 78.2% (76.0%–80.2%) | 0.485 (0.444–0.528) | 66.5% (64.6%–68.5%) |

*Reference standard: absolute CP ≥25%. Candidate stop was the index-test-negative classification.*

Supplementary Table S2. Performance of all 200 candidate warm composite rules

| **Rule ID** | **MCA threshold (%)** | **Minimum warm SPV threshold (°/s)** | **Spontaneous nystagmus condition** | **TP** | **FN** | **TN** | **FP** | **Sensitivity** | **Specificity** | **NPV** | **Candidate stop rate** | **CP ≥30% FN** | **CP ≥40% FN** | **Missed CP ≥25% per 1,000** | **Avoided cold irrigations per 1,000** | **Rule status** |
| --- | --- | --- | --- | --- | --- | --- | --- | --- | --- | --- | --- | --- | --- | --- | --- | --- |
| R001 | 5 | 5 | SPV = 0°/s | 1,939 | 13 | 278 | 3,207 | 99.3% | 8.0% | 95.5% | 5.4% | 3 | 0 | 2.4 | 107.0 | Meets primary sensitivity criterion |
| R002 | 5 | 6 | SPV = 0°/s | 1,941 | 11 | 266 | 3,219 | 99.4% | 7.6% | 96.0% | 5.1% | 2 | 0 | 2.0 | 101.9 | Meets primary sensitivity criterion |
| R003 | 5 | 8 | SPV = 0°/s | 1,943 | 9 | 244 | 3,241 | 99.5% | 7.0% | 96.4% | 4.7% | 2 | 0 | 1.7 | 93.1 | Meets primary sensitivity criterion |
| R004 | 5 | 10 | SPV = 0°/s | 1,946 | 6 | 217 | 3,268 | 99.7% | 6.2% | 97.3% | 4.1% | 0 | 0 | 1.1 | 82.0 | Meets primary sensitivity criterion |
| R005 | 5 | 11 | SPV = 0°/s | 1,946 | 6 | 205 | 3,280 | 99.7% | 5.9% | 97.2% | 3.9% | 0 | 0 | 1.1 | 77.6 | Meets primary sensitivity criterion |
| R006 | 7.5 | 5 | SPV = 0°/s | 1,929 | 23 | 415 | 3,070 | 98.8% | 11.9% | 94.7% | 8.1% | 6 | 1 | 4.2 | 161.1 | Meets primary sensitivity criterion |
| R007 | 7.5 | 6 | SPV = 0°/s | 1,931 | 21 | 403 | 3,082 | 98.9% | 11.6% | 95.0% | 7.8% | 5 | 1 | 3.9 | 156.0 | Meets primary sensitivity criterion |
| R008 | 7.5 | 8 | SPV = 0°/s | 1,937 | 15 | 355 | 3,130 | 99.2% | 10.2% | 95.9% | 6.8% | 4 | 1 | 2.8 | 136.1 | Meets primary sensitivity criterion |
| R009 | 7.5 | 10 | SPV = 0°/s | 1,942 | 10 | 317 | 3,168 | 99.5% | 9.1% | 96.9% | 6.0% | 1 | 0 | 1.8 | 120.3 | Meets primary sensitivity criterion |
| R010 | 7.5 | 11 | SPV = 0°/s | 1,942 | 10 | 305 | 3,180 | 99.5% | 8.8% | 96.8% | 5.8% | 1 | 0 | 1.8 | 115.9 | Meets primary sensitivity criterion |
| R011 | 10 | 5 | SPV = 0°/s | 1,920 | 32 | 542 | 2,943 | 98.4% | 15.6% | 94.4% | 10.6% | 10 | 2 | 5.9 | 211.1 | Meets primary sensitivity criterion |
| R012 | 10 | 6 | SPV = 0°/s | 1,926 | 26 | 515 | 2,970 | 98.7% | 14.8% | 95.2% | 10.0% | 7 | 1 | 4.8 | 199.0 | Meets primary sensitivity criterion |
| R013 | 10 | 8 | SPV = 0°/s | 1,932 | 20 | 467 | 3,018 | 99.0% | 13.4% | 95.9% | 9.0% | 6 | 1 | 3.7 | 179.1 | Meets primary sensitivity criterion |
| R014 | 10 | 10 | SPV = 0°/s | 1,939 | 13 | 423 | 3,062 | 99.3% | 12.1% | 97.0% | 8.0% | 3 | 0 | 2.4 | 160.4 | Meets primary sensitivity criterion |
| R015 | 10 | 11 | SPV = 0°/s | 1,941 | 11 | 403 | 3,082 | 99.4% | 11.6% | 97.3% | 7.6% | 2 | 0 | 2.0 | 152.3 | Meets primary sensitivity criterion |
| R016 | 12.5 | 5 | SPV = 0°/s | 1,916 | 36 | 676 | 2,809 | 98.2% | 19.4% | 94.9% | 13.1% | 13 | 2 | 6.6 | 261.9 | Meets primary sensitivity criterion |
| R017 | 12.5 | 6 | SPV = 0°/s | 1,922 | 30 | 649 | 2,836 | 98.5% | 18.6% | 95.6% | 12.5% | 10 | 1 | 5.5 | 249.8 | Meets primary sensitivity criterion |
| R018 | 12.5 | 8 | SPV = 0°/s | 1,929 | 23 | 585 | 2,900 | 98.8% | 16.8% | 96.2% | 11.2% | 8 | 1 | 4.2 | 223.7 | Meets primary sensitivity criterion |
| R019 | 12.5 | 10 | SPV = 0°/s | 1,936 | 16 | 531 | 2,954 | 99.2% | 15.2% | 97.1% | 10.1% | 5 | 0 | 2.9 | 201.2 | Meets primary sensitivity criterion |
| R020 | 12.5 | 11 | SPV = 0°/s | 1,938 | 14 | 501 | 2,984 | 99.3% | 14.4% | 97.3% | 9.5% | 4 | 0 | 2.6 | 189.4 | Meets primary sensitivity criterion |
| R021 | 15 | 5 | SPV = 0°/s | 1,907 | 45 | 827 | 2,658 | 97.7% | 23.7% | 94.8% | 16.0% | 20 | 4 | 8.3 | 320.8 | Meets primary sensitivity criterion |
| R022 | 15 | 6 | SPV = 0°/s | 1,914 | 38 | 785 | 2,700 | 98.1% | 22.5% | 95.4% | 15.1% | 16 | 3 | 7.0 | 302.7 | Meets primary sensitivity criterion |
| R023 | 15 | 8 | SPV = 0°/s | 1,922 | 30 | 707 | 2,778 | 98.5% | 20.3% | 95.9% | 13.6% | 13 | 3 | 5.5 | 271.1 | Meets primary sensitivity criterion |
| R024 | 15 | 10 | SPV = 0°/s | 1,930 | 22 | 627 | 2,858 | 98.9% | 18.0% | 96.6% | 11.9% | 10 | 2 | 4.0 | 238.7 | Meets primary sensitivity criterion |
| R025 | 15 | 11 | SPV = 0°/s | 1,932 | 20 | 597 | 2,888 | 99.0% | 17.1% | 96.8% | 11.3% | 9 | 2 | 3.7 | 227.0 | Meets primary sensitivity criterion |
| R026 | 17.5 | 5 | SPV = 0°/s | 1,901 | 51 | 923 | 2,562 | 97.4% | 26.5% | 94.8% | 17.9% | 23 | 4 | 9.4 | 358.3 | Below primary sensitivity criterion |
| R027 | 17.5 | 6 | SPV = 0°/s | 1,908 | 44 | 881 | 2,604 | 97.7% | 25.3% | 95.2% | 17.0% | 19 | 3 | 8.1 | 340.3 | Meets primary sensitivity criterion |
| R028 | 17.5 | 8 | SPV = 0°/s | 1,917 | 35 | 794 | 2,691 | 98.2% | 22.8% | 95.8% | 15.2% | 15 | 3 | 6.4 | 304.9 | Meets primary sensitivity criterion |
| R029 | 17.5 | 10 | SPV = 0°/s | 1,925 | 27 | 711 | 2,774 | 98.6% | 20.4% | 96.3% | 13.6% | 12 | 2 | 5.0 | 271.5 | Meets primary sensitivity criterion |
| R030 | 17.5 | 11 | SPV = 0°/s | 1,927 | 25 | 676 | 2,809 | 98.7% | 19.4% | 96.4% | 12.9% | 11 | 2 | 4.6 | 257.9 | Meets primary sensitivity criterion |
| R031 | 20 | 5 | SPV = 0°/s | 1,891 | 61 | 1,014 | 2,471 | 96.9% | 29.1% | 94.3% | 19.8% | 29 | 6 | 11.2 | 395.4 | Below primary sensitivity criterion |
| R032 | 20 | 6 | SPV = 0°/s | 1,898 | 54 | 972 | 2,513 | 97.2% | 27.9% | 94.7% | 18.9% | 25 | 5 | 9.9 | 377.4 | Below primary sensitivity criterion |
| R033 | 20 | 8 | SPV = 0°/s | 1,910 | 42 | 876 | 2,609 | 97.8% | 25.1% | 95.4% | 16.9% | 18 | 4 | 7.7 | 337.7 | Meets primary sensitivity criterion |
| R034 | 20 | 10 | SPV = 0°/s | 1,920 | 32 | 791 | 2,694 | 98.4% | 22.7% | 96.1% | 15.1% | 14 | 3 | 5.9 | 302.7 | Meets primary sensitivity criterion |
| R035 | 20 | 11 | SPV = 0°/s | 1,924 | 28 | 748 | 2,737 | 98.6% | 21.5% | 96.4% | 14.3% | 13 | 3 | 5.1 | 285.5 | Meets primary sensitivity criterion |
| R036 | 22.5 | 5 | SPV = 0°/s | 1,861 | 91 | 1,139 | 2,346 | 95.3% | 32.7% | 92.6% | 22.6% | 46 | 10 | 16.7 | 452.5 | Below primary sensitivity criterion |
| R037 | 22.5 | 6 | SPV = 0°/s | 1,871 | 81 | 1,087 | 2,398 | 95.9% | 31.2% | 93.1% | 21.5% | 40 | 8 | 14.9 | 429.6 | Below primary sensitivity criterion |
| R038 | 22.5 | 8 | SPV = 0°/s | 1,892 | 60 | 978 | 2,507 | 96.9% | 28.1% | 94.2% | 19.1% | 27 | 4 | 11.0 | 381.8 | Below primary sensitivity criterion |
| R039 | 22.5 | 10 | SPV = 0°/s | 1,904 | 48 | 875 | 2,610 | 97.5% | 25.1% | 94.8% | 17.0% | 22 | 3 | 8.8 | 339.5 | Meets primary sensitivity criterion |
| R040 | 22.5 | 11 | SPV = 0°/s | 1,910 | 42 | 832 | 2,653 | 97.8% | 23.9% | 95.2% | 16.1% | 20 | 3 | 7.7 | 321.5 | Meets primary sensitivity criterion |
| R041 | 25 | 5 | SPV = 0°/s | 1,845 | 107 | 1,215 | 2,270 | 94.5% | 34.9% | 91.9% | 24.3% | 51 | 11 | 19.7 | 486.3 | Below primary sensitivity criterion |
| R042 | 25 | 6 | SPV = 0°/s | 1,855 | 97 | 1,163 | 2,322 | 95.0% | 33.4% | 92.3% | 23.2% | 45 | 9 | 17.8 | 463.5 | Below primary sensitivity criterion |
| R043 | 25 | 8 | SPV = 0°/s | 1,879 | 73 | 1,045 | 2,440 | 96.3% | 30.0% | 93.5% | 20.6% | 32 | 5 | 13.4 | 411.3 | Below primary sensitivity criterion |
| R044 | 25 | 10 | SPV = 0°/s | 1,891 | 61 | 938 | 2,547 | 96.9% | 26.9% | 93.9% | 18.4% | 27 | 4 | 11.2 | 367.5 | Below primary sensitivity criterion |
| R045 | 25 | 11 | SPV = 0°/s | 1,899 | 53 | 888 | 2,597 | 97.3% | 25.5% | 94.4% | 17.3% | 24 | 4 | 9.7 | 346.1 | Below primary sensitivity criterion |
| R046 | 30 | 5 | SPV = 0°/s | 1,787 | 165 | 1,381 | 2,104 | 91.5% | 39.6% | 89.3% | 28.4% | 78 | 21 | 30.3 | 568.7 | Below primary sensitivity criterion |
| R047 | 30 | 6 | SPV = 0°/s | 1,803 | 149 | 1,310 | 2,175 | 92.4% | 37.6% | 89.8% | 26.8% | 69 | 19 | 27.4 | 536.7 | Below primary sensitivity criterion |
| R048 | 30 | 8 | SPV = 0°/s | 1,835 | 117 | 1,178 | 2,307 | 94.0% | 33.8% | 91.0% | 23.8% | 49 | 10 | 21.5 | 476.4 | Below primary sensitivity criterion |
| R049 | 30 | 10 | SPV = 0°/s | 1,852 | 100 | 1,056 | 2,429 | 94.9% | 30.3% | 91.3% | 21.3% | 40 | 8 | 18.4 | 425.2 | Below primary sensitivity criterion |
| R050 | 30 | 11 | SPV = 0°/s | 1,861 | 91 | 992 | 2,493 | 95.3% | 28.5% | 91.6% | 19.9% | 37 | 8 | 16.7 | 398.4 | Below primary sensitivity criterion |
| R051 | 5 | 5 | SPV <2°/s | 1,934 | 18 | 472 | 3,013 | 99.1% | 13.5% | 96.3% | 9.0% | 6 | 0 | 3.3 | 180.2 | Meets primary sensitivity criterion |
| R052 | 5 | 6 | SPV <2°/s | 1,936 | 16 | 451 | 3,034 | 99.2% | 12.9% | 96.6% | 8.6% | 5 | 0 | 2.9 | 171.8 | Meets primary sensitivity criterion |
| R053 | 5 | 8 | SPV <2°/s | 1,938 | 14 | 418 | 3,067 | 99.3% | 12.0% | 96.8% | 7.9% | 5 | 0 | 2.6 | 158.9 | Meets primary sensitivity criterion |
| R054 | 5 | 10 | SPV <2°/s | 1,943 | 9 | 379 | 3,106 | 99.5% | 10.9% | 97.7% | 7.1% | 2 | 0 | 1.7 | 142.7 | Meets primary sensitivity criterion |
| R055 | 5 | 11 | SPV <2°/s | 1,944 | 8 | 357 | 3,128 | 99.6% | 10.2% | 97.8% | 6.7% | 1 | 0 | 1.5 | 134.3 | Meets primary sensitivity criterion |
| R056 | 7.5 | 5 | SPV <2°/s | 1,922 | 30 | 695 | 2,790 | 98.5% | 19.9% | 95.9% | 13.3% | 9 | 1 | 5.5 | 266.7 | Meets primary sensitivity criterion |
| R057 | 7.5 | 6 | SPV <2°/s | 1,924 | 28 | 674 | 2,811 | 98.6% | 19.3% | 96.0% | 12.9% | 8 | 1 | 5.1 | 258.2 | Meets primary sensitivity criterion |
| R058 | 7.5 | 8 | SPV <2°/s | 1,931 | 21 | 605 | 2,880 | 98.9% | 17.4% | 96.6% | 11.5% | 7 | 1 | 3.9 | 230.3 | Meets primary sensitivity criterion |
| R059 | 7.5 | 10 | SPV <2°/s | 1,938 | 14 | 552 | 2,933 | 99.3% | 15.8% | 97.5% | 10.4% | 3 | 0 | 2.6 | 208.2 | Meets primary sensitivity criterion |
| R060 | 7.5 | 11 | SPV <2°/s | 1,939 | 13 | 530 | 2,955 | 99.3% | 15.2% | 97.6% | 10.0% | 2 | 0 | 2.4 | 199.7 | Meets primary sensitivity criterion |
| R061 | 10 | 5 | SPV <2°/s | 1,909 | 43 | 898 | 2,587 | 97.8% | 25.8% | 95.4% | 17.3% | 16 | 2 | 7.9 | 346.1 | Meets primary sensitivity criterion |
| R062 | 10 | 6 | SPV <2°/s | 1,917 | 35 | 850 | 2,635 | 98.2% | 24.4% | 96.0% | 16.3% | 11 | 1 | 6.4 | 325.5 | Meets primary sensitivity criterion |
| R063 | 10 | 8 | SPV <2°/s | 1,924 | 28 | 781 | 2,704 | 98.6% | 22.4% | 96.5% | 14.9% | 10 | 1 | 5.1 | 297.6 | Meets primary sensitivity criterion |
| R064 | 10 | 10 | SPV <2°/s | 1,934 | 18 | 717 | 2,768 | 99.1% | 20.6% | 97.6% | 13.5% | 6 | 0 | 3.3 | 270.4 | Meets primary sensitivity criterion |
| R065 | 10 | 11 | SPV <2°/s | 1,937 | 15 | 684 | 2,801 | 99.2% | 19.6% | 97.9% | 12.9% | 4 | 0 | 2.8 | 257.1 | Meets primary sensitivity criterion |
| R066 | 12.5 | 5 | SPV <2°/s | 1,900 | 52 | 1,095 | 2,390 | 97.3% | 31.4% | 95.5% | 21.1% | 22 | 3 | 9.6 | 421.9 | Below primary sensitivity criterion |
| R067 | 12.5 | 6 | SPV <2°/s | 1,908 | 44 | 1,047 | 2,438 | 97.7% | 30.0% | 96.0% | 20.1% | 17 | 2 | 8.1 | 401.3 | Meets primary sensitivity criterion |
| R068 | 12.5 | 8 | SPV <2°/s | 1,918 | 34 | 958 | 2,527 | 98.3% | 27.5% | 96.6% | 18.2% | 13 | 2 | 6.3 | 364.9 | Meets primary sensitivity criterion |
| R069 | 12.5 | 10 | SPV <2°/s | 1,928 | 24 | 876 | 2,609 | 98.8% | 25.1% | 97.3% | 16.6% | 9 | 1 | 4.4 | 331.1 | Meets primary sensitivity criterion |
| R070 | 12.5 | 11 | SPV <2°/s | 1,932 | 20 | 831 | 2,654 | 99.0% | 23.8% | 97.6% | 15.7% | 6 | 0 | 3.7 | 313.0 | Meets primary sensitivity criterion |
| R071 | 15 | 5 | SPV <2°/s | 1,884 | 68 | 1,311 | 2,174 | 96.5% | 37.6% | 95.1% | 25.4% | 34 | 5 | 12.5 | 507.3 | Below primary sensitivity criterion |
| R072 | 15 | 6 | SPV <2°/s | 1,895 | 57 | 1,239 | 2,246 | 97.1% | 35.6% | 95.6% | 23.8% | 27 | 4 | 10.5 | 476.7 | Below primary sensitivity criterion |
| R073 | 15 | 8 | SPV <2°/s | 1,907 | 45 | 1,127 | 2,358 | 97.7% | 32.3% | 96.2% | 21.6% | 22 | 4 | 8.3 | 431.1 | Meets primary sensitivity criterion |
| R074 | 15 | 10 | SPV <2°/s | 1,919 | 33 | 1,009 | 2,476 | 98.3% | 29.0% | 96.8% | 19.2% | 17 | 3 | 6.1 | 383.3 | Meets primary sensitivity criterion |
| R075 | 15 | 11 | SPV <2°/s | 1,923 | 29 | 964 | 2,521 | 98.5% | 27.7% | 97.1% | 18.3% | 14 | 2 | 5.3 | 365.3 | Meets primary sensitivity criterion |
| R076 | 17.5 | 5 | SPV <2°/s | 1,871 | 81 | 1,458 | 2,027 | 95.9% | 41.8% | 94.7% | 28.3% | 42 | 6 | 14.9 | 566.1 | Below primary sensitivity criterion |
| R077 | 17.5 | 6 | SPV <2°/s | 1,882 | 70 | 1,386 | 2,099 | 96.4% | 39.8% | 95.2% | 26.8% | 35 | 5 | 12.9 | 535.6 | Below primary sensitivity criterion |
| R078 | 17.5 | 8 | SPV <2°/s | 1,898 | 54 | 1,262 | 2,223 | 97.2% | 36.2% | 95.9% | 24.2% | 27 | 4 | 9.9 | 484.1 | Below primary sensitivity criterion |
| R079 | 17.5 | 10 | SPV <2°/s | 1,911 | 41 | 1,134 | 2,351 | 97.9% | 32.5% | 96.5% | 21.6% | 22 | 3 | 7.5 | 432.2 | Meets primary sensitivity criterion |
| R080 | 17.5 | 11 | SPV <2°/s | 1,915 | 37 | 1,078 | 2,407 | 98.1% | 30.9% | 96.7% | 20.5% | 19 | 2 | 6.8 | 410.2 | Meets primary sensitivity criterion |
| R081 | 20 | 5 | SPV <2°/s | 1,851 | 101 | 1,616 | 1,869 | 94.8% | 46.4% | 94.1% | 31.6% | 50 | 9 | 18.6 | 631.6 | Below primary sensitivity criterion |
| R082 | 20 | 6 | SPV <2°/s | 1,862 | 90 | 1,544 | 1,941 | 95.4% | 44.3% | 94.5% | 30.1% | 43 | 8 | 16.6 | 601.1 | Below primary sensitivity criterion |
| R083 | 20 | 8 | SPV <2°/s | 1,885 | 67 | 1,400 | 2,085 | 96.6% | 40.2% | 95.4% | 27.0% | 31 | 6 | 12.3 | 539.6 | Below primary sensitivity criterion |
| R084 | 20 | 10 | SPV <2°/s | 1,900 | 52 | 1,263 | 2,222 | 97.3% | 36.2% | 96.0% | 24.2% | 25 | 5 | 9.6 | 483.7 | Below primary sensitivity criterion |
| R085 | 20 | 11 | SPV <2°/s | 1,906 | 46 | 1,195 | 2,290 | 97.6% | 34.3% | 96.3% | 22.8% | 22 | 4 | 8.5 | 456.5 | Meets primary sensitivity criterion |
| R086 | 22.5 | 5 | SPV <2°/s | 1,808 | 144 | 1,808 | 1,677 | 92.6% | 51.9% | 92.6% | 35.9% | 72 | 13 | 26.5 | 718.0 | Below primary sensitivity criterion |
| R087 | 22.5 | 6 | SPV <2°/s | 1,827 | 125 | 1,725 | 1,760 | 93.6% | 49.5% | 93.2% | 34.0% | 61 | 11 | 23.0 | 680.5 | Below primary sensitivity criterion |
| R088 | 22.5 | 8 | SPV <2°/s | 1,863 | 89 | 1,556 | 1,929 | 95.4% | 44.6% | 94.6% | 30.3% | 41 | 6 | 16.4 | 605.1 | Below primary sensitivity criterion |
| R089 | 22.5 | 10 | SPV <2°/s | 1,881 | 71 | 1,394 | 2,091 | 96.4% | 40.0% | 95.2% | 26.9% | 34 | 5 | 13.1 | 538.9 | Below primary sensitivity criterion |
| R090 | 22.5 | 11 | SPV <2°/s | 1,890 | 62 | 1,321 | 2,164 | 96.8% | 37.9% | 95.5% | 25.4% | 30 | 4 | 11.4 | 508.7 | Below primary sensitivity criterion |
| R091 | 25 | 5 | SPV <2°/s | 1,781 | 171 | 1,913 | 1,572 | 91.2% | 54.9% | 91.8% | 38.3% | 82 | 15 | 31.5 | 766.6 | Below primary sensitivity criterion |
| R092 | 25 | 6 | SPV <2°/s | 1,800 | 152 | 1,830 | 1,655 | 92.2% | 52.5% | 92.3% | 36.5% | 71 | 13 | 28.0 | 729.1 | Below primary sensitivity criterion |
| R093 | 25 | 8 | SPV <2°/s | 1,840 | 112 | 1,648 | 1,837 | 94.3% | 47.3% | 93.6% | 32.4% | 50 | 8 | 20.6 | 647.4 | Below primary sensitivity criterion |
| R094 | 25 | 10 | SPV <2°/s | 1,858 | 94 | 1,480 | 2,005 | 95.2% | 42.5% | 94.0% | 28.9% | 43 | 7 | 17.3 | 579.0 | Below primary sensitivity criterion |
| R095 | 25 | 11 | SPV <2°/s | 1,869 | 83 | 1,398 | 2,087 | 95.7% | 40.1% | 94.4% | 27.2% | 38 | 6 | 15.3 | 544.8 | Below primary sensitivity criterion |
| R096 | 30 | 5 | SPV <2°/s | 1,681 | 271 | 2,151 | 1,334 | 86.1% | 61.7% | 88.8% | 44.5% | 140 | 29 | 49.8 | 890.9 | Below primary sensitivity criterion |
| R097 | 30 | 6 | SPV <2°/s | 1,710 | 242 | 2,041 | 1,444 | 87.6% | 58.6% | 89.4% | 42.0% | 122 | 24 | 44.5 | 839.8 | Below primary sensitivity criterion |
| R098 | 30 | 8 | SPV <2°/s | 1,763 | 189 | 1,840 | 1,645 | 90.3% | 52.8% | 90.7% | 37.3% | 91 | 14 | 34.8 | 746.4 | Below primary sensitivity criterion |
| R099 | 30 | 10 | SPV <2°/s | 1,799 | 153 | 1,648 | 1,837 | 92.2% | 47.3% | 91.5% | 33.1% | 69 | 12 | 28.1 | 662.5 | Below primary sensitivity criterion |
| R100 | 30 | 11 | SPV <2°/s | 1,812 | 140 | 1,547 | 1,938 | 92.8% | 44.4% | 91.7% | 31.0% | 64 | 11 | 25.7 | 620.6 | Below primary sensitivity criterion |
| R101 | 5 | 5 | SPV <4°/s | 1,930 | 22 | 563 | 2,922 | 98.9% | 16.2% | 96.2% | 10.8% | 8 | 0 | 4.0 | 215.2 | Meets primary sensitivity criterion |
| R102 | 5 | 6 | SPV <4°/s | 1,932 | 20 | 541 | 2,944 | 99.0% | 15.5% | 96.4% | 10.3% | 7 | 0 | 3.7 | 206.4 | Meets primary sensitivity criterion |
| R103 | 5 | 8 | SPV <4°/s | 1,935 | 17 | 504 | 2,981 | 99.1% | 14.5% | 96.7% | 9.6% | 6 | 0 | 3.1 | 191.6 | Meets primary sensitivity criterion |
| R104 | 5 | 10 | SPV <4°/s | 1,941 | 11 | 456 | 3,029 | 99.4% | 13.1% | 97.6% | 8.6% | 3 | 0 | 2.0 | 171.8 | Meets primary sensitivity criterion |
| R105 | 5 | 11 | SPV <4°/s | 1,942 | 10 | 427 | 3,058 | 99.5% | 12.3% | 97.7% | 8.0% | 2 | 0 | 1.8 | 160.8 | Meets primary sensitivity criterion |
| R106 | 7.5 | 5 | SPV <4°/s | 1,916 | 36 | 823 | 2,662 | 98.2% | 23.6% | 95.8% | 15.8% | 13 | 2 | 6.6 | 316.0 | Meets primary sensitivity criterion |
| R107 | 7.5 | 6 | SPV <4°/s | 1,918 | 34 | 801 | 2,684 | 98.3% | 23.0% | 95.9% | 15.4% | 12 | 2 | 6.3 | 307.2 | Meets primary sensitivity criterion |
| R108 | 7.5 | 8 | SPV <4°/s | 1,926 | 26 | 722 | 2,763 | 98.7% | 20.7% | 96.5% | 13.8% | 10 | 2 | 4.8 | 275.2 | Meets primary sensitivity criterion |
| R109 | 7.5 | 10 | SPV <4°/s | 1,935 | 17 | 660 | 2,825 | 99.1% | 18.9% | 97.5% | 12.5% | 5 | 0 | 3.1 | 249.0 | Meets primary sensitivity criterion |
| R110 | 7.5 | 11 | SPV <4°/s | 1,936 | 16 | 631 | 2,854 | 99.2% | 18.1% | 97.5% | 11.9% | 4 | 0 | 2.9 | 238.0 | Meets primary sensitivity criterion |
| R111 | 10 | 5 | SPV <4°/s | 1,900 | 52 | 1,061 | 2,424 | 97.3% | 30.4% | 95.3% | 20.5% | 20 | 3 | 9.6 | 409.4 | Below primary sensitivity criterion |
| R112 | 10 | 6 | SPV <4°/s | 1,908 | 44 | 1,009 | 2,476 | 97.7% | 29.0% | 95.8% | 19.4% | 15 | 2 | 8.1 | 387.3 | Meets primary sensitivity criterion |
| R113 | 10 | 8 | SPV <4°/s | 1,916 | 36 | 930 | 2,555 | 98.2% | 26.7% | 96.3% | 17.8% | 13 | 2 | 6.6 | 355.3 | Meets primary sensitivity criterion |
| R114 | 10 | 10 | SPV <4°/s | 1,928 | 24 | 854 | 2,631 | 98.8% | 24.5% | 97.3% | 16.1% | 8 | 0 | 4.4 | 323.0 | Meets primary sensitivity criterion |
| R115 | 10 | 11 | SPV <4°/s | 1,932 | 20 | 813 | 2,672 | 99.0% | 23.3% | 97.6% | 15.3% | 6 | 0 | 3.7 | 306.4 | Meets primary sensitivity criterion |
| R116 | 12.5 | 5 | SPV <4°/s | 1,887 | 65 | 1,294 | 2,191 | 96.7% | 37.1% | 95.2% | 25.0% | 28 | 4 | 12.0 | 499.9 | Below primary sensitivity criterion |
| R117 | 12.5 | 6 | SPV <4°/s | 1,895 | 57 | 1,242 | 2,243 | 97.1% | 35.6% | 95.6% | 23.9% | 23 | 3 | 10.5 | 477.8 | Below primary sensitivity criterion |
| R118 | 12.5 | 8 | SPV <4°/s | 1,906 | 46 | 1,138 | 2,347 | 97.6% | 32.7% | 96.1% | 21.8% | 18 | 3 | 8.5 | 435.5 | Meets primary sensitivity criterion |
| R119 | 12.5 | 10 | SPV <4°/s | 1,920 | 32 | 1,043 | 2,442 | 98.4% | 29.9% | 97.0% | 19.8% | 11 | 1 | 5.9 | 395.4 | Meets primary sensitivity criterion |
| R120 | 12.5 | 11 | SPV <4°/s | 1,925 | 27 | 987 | 2,498 | 98.6% | 28.3% | 97.3% | 18.6% | 8 | 0 | 5.0 | 373.0 | Meets primary sensitivity criterion |
| R121 | 15 | 5 | SPV <4°/s | 1,865 | 87 | 1,537 | 1,948 | 95.5% | 44.1% | 94.6% | 29.9% | 45 | 7 | 16.0 | 597.4 | Below primary sensitivity criterion |
| R122 | 15 | 6 | SPV <4°/s | 1,877 | 75 | 1,459 | 2,026 | 96.2% | 41.9% | 95.1% | 28.2% | 37 | 6 | 13.8 | 564.3 | Below primary sensitivity criterion |
| R123 | 15 | 8 | SPV <4°/s | 1,892 | 60 | 1,330 | 2,155 | 96.9% | 38.2% | 95.7% | 25.6% | 29 | 6 | 11.0 | 511.3 | Below primary sensitivity criterion |
| R124 | 15 | 10 | SPV <4°/s | 1,910 | 42 | 1,193 | 2,292 | 97.8% | 34.2% | 96.6% | 22.7% | 19 | 3 | 7.7 | 454.3 | Meets primary sensitivity criterion |
| R125 | 15 | 11 | SPV <4°/s | 1,915 | 37 | 1,137 | 2,348 | 98.1% | 32.6% | 96.8% | 21.6% | 16 | 2 | 6.8 | 431.9 | Meets primary sensitivity criterion |
| R126 | 17.5 | 5 | SPV <4°/s | 1,850 | 102 | 1,718 | 1,767 | 94.8% | 49.3% | 94.4% | 33.5% | 54 | 8 | 18.8 | 669.5 | Below primary sensitivity criterion |
| R127 | 17.5 | 6 | SPV <4°/s | 1,862 | 90 | 1,640 | 1,845 | 95.4% | 47.1% | 94.8% | 31.8% | 46 | 7 | 16.6 | 636.4 | Below primary sensitivity criterion |
| R128 | 17.5 | 8 | SPV <4°/s | 1,881 | 71 | 1,497 | 1,988 | 96.4% | 43.0% | 95.5% | 28.8% | 35 | 6 | 13.1 | 576.8 | Below primary sensitivity criterion |
| R129 | 17.5 | 10 | SPV <4°/s | 1,901 | 51 | 1,348 | 2,137 | 97.4% | 38.7% | 96.4% | 25.7% | 24 | 3 | 9.4 | 514.6 | Below primary sensitivity criterion |
| R130 | 17.5 | 11 | SPV <4°/s | 1,906 | 46 | 1,281 | 2,204 | 97.6% | 36.8% | 96.5% | 24.4% | 21 | 2 | 8.5 | 488.1 | Final selected rule |
| R131 | 20 | 5 | SPV <4°/s | 1,826 | 126 | 1,899 | 1,586 | 93.5% | 54.5% | 93.8% | 37.2% | 64 | 11 | 23.2 | 744.9 | Below primary sensitivity criterion |
| R132 | 20 | 6 | SPV <4°/s | 1,838 | 114 | 1,821 | 1,664 | 94.2% | 52.3% | 94.1% | 35.6% | 56 | 10 | 21.0 | 711.8 | Below primary sensitivity criterion |
| R133 | 20 | 8 | SPV <4°/s | 1,865 | 87 | 1,656 | 1,829 | 95.5% | 47.5% | 95.0% | 32.1% | 40 | 8 | 16.0 | 641.2 | Below primary sensitivity criterion |
| R134 | 20 | 10 | SPV <4°/s | 1,887 | 65 | 1,496 | 1,989 | 96.7% | 42.9% | 95.8% | 28.7% | 28 | 5 | 12.0 | 574.2 | Below primary sensitivity criterion |
| R135 | 20 | 11 | SPV <4°/s | 1,894 | 58 | 1,416 | 2,069 | 97.0% | 40.6% | 96.1% | 27.1% | 25 | 4 | 10.7 | 542.2 | Below primary sensitivity criterion |
| R136 | 22.5 | 5 | SPV <4°/s | 1,773 | 179 | 2,140 | 1,345 | 90.8% | 61.4% | 92.3% | 42.7% | 89 | 15 | 32.9 | 853.0 | Below primary sensitivity criterion |
| R137 | 22.5 | 6 | SPV <4°/s | 1,794 | 158 | 2,047 | 1,438 | 91.9% | 58.7% | 92.8% | 40.6% | 77 | 13 | 29.1 | 811.1 | Below primary sensitivity criterion |
| R138 | 22.5 | 8 | SPV <4°/s | 1,837 | 115 | 1,851 | 1,634 | 94.1% | 53.1% | 94.2% | 36.2% | 51 | 8 | 21.2 | 723.2 | Below primary sensitivity criterion |
| R139 | 22.5 | 10 | SPV <4°/s | 1,863 | 89 | 1,664 | 1,821 | 95.4% | 47.7% | 94.9% | 32.2% | 38 | 5 | 16.4 | 644.8 | Below primary sensitivity criterion |
| R140 | 22.5 | 11 | SPV <4°/s | 1,873 | 79 | 1,574 | 1,911 | 96.0% | 45.2% | 95.2% | 30.4% | 34 | 4 | 14.5 | 608.1 | Below primary sensitivity criterion |
| R141 | 25 | 5 | SPV <4°/s | 1,743 | 209 | 2,267 | 1,218 | 89.3% | 65.1% | 91.6% | 45.5% | 99 | 17 | 38.4 | 910.8 | Below primary sensitivity criterion |
| R142 | 25 | 6 | SPV <4°/s | 1,764 | 188 | 2,174 | 1,311 | 90.4% | 62.4% | 92.0% | 43.4% | 87 | 15 | 34.6 | 868.9 | Below primary sensitivity criterion |
| R143 | 25 | 8 | SPV <4°/s | 1,811 | 141 | 1,965 | 1,520 | 92.8% | 56.4% | 93.3% | 38.7% | 60 | 10 | 25.9 | 774.7 | Below primary sensitivity criterion |
| R144 | 25 | 10 | SPV <4°/s | 1,838 | 114 | 1,772 | 1,713 | 94.2% | 50.8% | 94.0% | 34.7% | 47 | 7 | 21.0 | 693.8 | Below primary sensitivity criterion |
| R145 | 25 | 11 | SPV <4°/s | 1,851 | 101 | 1,673 | 1,812 | 94.8% | 48.0% | 94.3% | 32.6% | 42 | 6 | 18.6 | 652.6 | Below primary sensitivity criterion |
| R146 | 30 | 5 | SPV <4°/s | 1,630 | 322 | 2,547 | 938 | 83.5% | 73.1% | 88.8% | 52.8% | 165 | 33 | 59.2 | 1055.4 | Below primary sensitivity criterion |
| R147 | 30 | 6 | SPV <4°/s | 1,661 | 291 | 2,422 | 1,063 | 85.1% | 69.5% | 89.3% | 49.9% | 146 | 28 | 53.5 | 998.0 | Below primary sensitivity criterion |
| R148 | 30 | 8 | SPV <4°/s | 1,725 | 227 | 2,189 | 1,296 | 88.4% | 62.8% | 90.6% | 44.4% | 107 | 18 | 41.8 | 888.7 | Below primary sensitivity criterion |
| R149 | 30 | 10 | SPV <4°/s | 1,771 | 181 | 1,967 | 1,518 | 90.7% | 56.4% | 91.6% | 39.5% | 78 | 13 | 33.3 | 790.1 | Below primary sensitivity criterion |
| R150 | 30 | 11 | SPV <4°/s | 1,786 | 166 | 1,848 | 1,637 | 91.5% | 53.0% | 91.8% | 37.0% | 73 | 12 | 30.5 | 740.8 | Below primary sensitivity criterion |
| R151 | 5 | 5 | SPV ≤4°/s | 1,929 | 23 | 567 | 2,918 | 98.8% | 16.3% | 96.1% | 10.9% | 8 | 0 | 4.2 | 217.0 | Meets primary sensitivity criterion |
| R152 | 5 | 6 | SPV ≤4°/s | 1,931 | 21 | 545 | 2,940 | 98.9% | 15.6% | 96.3% | 10.4% | 7 | 0 | 3.9 | 208.2 | Meets primary sensitivity criterion |
| R153 | 5 | 8 | SPV ≤4°/s | 1,934 | 18 | 508 | 2,977 | 99.1% | 14.6% | 96.6% | 9.7% | 6 | 0 | 3.3 | 193.5 | Meets primary sensitivity criterion |
| R154 | 5 | 10 | SPV ≤4°/s | 1,940 | 12 | 459 | 3,026 | 99.4% | 13.2% | 97.5% | 8.7% | 3 | 0 | 2.2 | 173.3 | Meets primary sensitivity criterion |
| R155 | 5 | 11 | SPV ≤4°/s | 1,941 | 11 | 429 | 3,056 | 99.4% | 12.3% | 97.5% | 8.1% | 2 | 0 | 2.0 | 161.9 | Meets primary sensitivity criterion |
| R156 | 7.5 | 5 | SPV ≤4°/s | 1,915 | 37 | 831 | 2,654 | 98.1% | 23.8% | 95.7% | 16.0% | 13 | 2 | 6.8 | 319.3 | Meets primary sensitivity criterion |
| R157 | 7.5 | 6 | SPV ≤4°/s | 1,917 | 35 | 809 | 2,676 | 98.2% | 23.2% | 95.9% | 15.5% | 12 | 2 | 6.4 | 310.5 | Meets primary sensitivity criterion |
| R158 | 7.5 | 8 | SPV ≤4°/s | 1,925 | 27 | 729 | 2,756 | 98.6% | 20.9% | 96.4% | 13.9% | 10 | 2 | 5.0 | 278.1 | Meets primary sensitivity criterion |
| R159 | 7.5 | 10 | SPV ≤4°/s | 1,934 | 18 | 666 | 2,819 | 99.1% | 19.1% | 97.4% | 12.6% | 5 | 0 | 3.3 | 251.6 | Meets primary sensitivity criterion |
| R160 | 7.5 | 11 | SPV ≤4°/s | 1,935 | 17 | 636 | 2,849 | 99.1% | 18.2% | 97.4% | 12.0% | 4 | 0 | 3.1 | 240.2 | Meets primary sensitivity criterion |
| R161 | 10 | 5 | SPV ≤4°/s | 1,899 | 53 | 1,072 | 2,413 | 97.3% | 30.8% | 95.3% | 20.7% | 20 | 3 | 9.7 | 413.8 | Below primary sensitivity criterion |
| R162 | 10 | 6 | SPV ≤4°/s | 1,907 | 45 | 1,019 | 2,466 | 97.7% | 29.2% | 95.8% | 19.6% | 15 | 2 | 8.3 | 391.4 | Meets primary sensitivity criterion |
| R163 | 10 | 8 | SPV ≤4°/s | 1,915 | 37 | 939 | 2,546 | 98.1% | 26.9% | 96.2% | 18.0% | 13 | 2 | 6.8 | 359.0 | Meets primary sensitivity criterion |
| R164 | 10 | 10 | SPV ≤4°/s | 1,927 | 25 | 862 | 2,623 | 98.7% | 24.7% | 97.2% | 16.3% | 8 | 0 | 4.6 | 326.3 | Meets primary sensitivity criterion |
| R165 | 10 | 11 | SPV ≤4°/s | 1,931 | 21 | 820 | 2,665 | 98.9% | 23.5% | 97.5% | 15.5% | 6 | 0 | 3.9 | 309.4 | Meets primary sensitivity criterion |
| R166 | 12.5 | 5 | SPV ≤4°/s | 1,886 | 66 | 1,308 | 2,177 | 96.6% | 37.5% | 95.2% | 25.3% | 28 | 4 | 12.1 | 505.4 | Below primary sensitivity criterion |
| R167 | 12.5 | 6 | SPV ≤4°/s | 1,894 | 58 | 1,255 | 2,230 | 97.0% | 36.0% | 95.6% | 24.1% | 23 | 3 | 10.7 | 483.0 | Below primary sensitivity criterion |
| R168 | 12.5 | 8 | SPV ≤4°/s | 1,905 | 47 | 1,149 | 2,336 | 97.6% | 33.0% | 96.1% | 22.0% | 18 | 3 | 8.6 | 439.9 | Meets primary sensitivity criterion |
| R169 | 12.5 | 10 | SPV ≤4°/s | 1,919 | 33 | 1,052 | 2,433 | 98.3% | 30.2% | 97.0% | 20.0% | 11 | 1 | 6.1 | 399.1 | Meets primary sensitivity criterion |
| R170 | 12.5 | 11 | SPV ≤4°/s | 1,924 | 28 | 995 | 2,490 | 98.6% | 28.6% | 97.3% | 18.8% | 8 | 0 | 5.1 | 376.3 | Meets primary sensitivity criterion |
| R171 | 15 | 5 | SPV ≤4°/s | 1,864 | 88 | 1,554 | 1,931 | 95.5% | 44.6% | 94.6% | 30.2% | 45 | 7 | 16.2 | 604.0 | Below primary sensitivity criterion |
| R172 | 15 | 6 | SPV ≤4°/s | 1,876 | 76 | 1,475 | 2,010 | 96.1% | 42.3% | 95.1% | 28.5% | 37 | 6 | 14.0 | 570.5 | Below primary sensitivity criterion |
| R173 | 15 | 8 | SPV ≤4°/s | 1,891 | 61 | 1,344 | 2,141 | 96.9% | 38.6% | 95.7% | 25.8% | 29 | 6 | 11.2 | 516.8 | BSA-like reference rule |
| R174 | 15 | 10 | SPV ≤4°/s | 1,909 | 43 | 1,205 | 2,280 | 97.8% | 34.6% | 96.6% | 23.0% | 19 | 3 | 7.9 | 459.1 | Meets primary sensitivity criterion |
| R175 | 15 | 11 | SPV ≤4°/s | 1,914 | 38 | 1,148 | 2,337 | 98.1% | 32.9% | 96.8% | 21.8% | 16 | 2 | 7.0 | 436.3 | Meets primary sensitivity criterion |
| R176 | 17.5 | 5 | SPV ≤4°/s | 1,849 | 103 | 1,735 | 1,750 | 94.7% | 49.8% | 94.4% | 33.8% | 54 | 8 | 18.9 | 676.1 | Below primary sensitivity criterion |
| R177 | 17.5 | 6 | SPV ≤4°/s | 1,861 | 91 | 1,656 | 1,829 | 95.3% | 47.5% | 94.8% | 32.1% | 46 | 7 | 16.7 | 642.6 | Below primary sensitivity criterion |
| R178 | 17.5 | 8 | SPV ≤4°/s | 1,880 | 72 | 1,511 | 1,974 | 96.3% | 43.4% | 95.5% | 29.1% | 35 | 6 | 13.2 | 582.3 | Below primary sensitivity criterion |
| R179 | 17.5 | 10 | SPV ≤4°/s | 1,900 | 52 | 1,360 | 2,125 | 97.3% | 39.0% | 96.3% | 26.0% | 24 | 3 | 9.6 | 519.4 | Below primary sensitivity criterion |
| R180 | 17.5 | 11 | SPV ≤4°/s | 1,905 | 47 | 1,292 | 2,193 | 97.6% | 37.1% | 96.5% | 24.6% | 21 | 2 | 8.6 | 492.6 | Meets primary sensitivity criterion |
| R181 | 20 | 5 | SPV ≤4°/s | 1,824 | 128 | 1,920 | 1,565 | 93.4% | 55.1% | 93.8% | 37.7% | 65 | 12 | 23.5 | 753.4 | Below primary sensitivity criterion |
| R182 | 20 | 6 | SPV ≤4°/s | 1,836 | 116 | 1,841 | 1,644 | 94.1% | 52.8% | 94.1% | 36.0% | 57 | 11 | 21.3 | 719.9 | Below primary sensitivity criterion |
| R183 | 20 | 8 | SPV ≤4°/s | 1,863 | 89 | 1,674 | 1,811 | 95.4% | 48.0% | 95.0% | 32.4% | 41 | 9 | 16.4 | 648.5 | Below primary sensitivity criterion |
| R184 | 20 | 10 | SPV ≤4°/s | 1,885 | 67 | 1,512 | 1,973 | 96.6% | 43.4% | 95.8% | 29.0% | 29 | 6 | 12.3 | 580.8 | Below primary sensitivity criterion |
| R185 | 20 | 11 | SPV ≤4°/s | 1,892 | 60 | 1,430 | 2,055 | 96.9% | 41.0% | 96.0% | 27.4% | 26 | 5 | 11.0 | 548.1 | Below primary sensitivity criterion |
| R186 | 22.5 | 5 | SPV ≤4°/s | 1,771 | 181 | 2,163 | 1,322 | 90.7% | 62.1% | 92.3% | 43.1% | 90 | 16 | 33.3 | 862.2 | Below primary sensitivity criterion |
| R187 | 22.5 | 6 | SPV ≤4°/s | 1,792 | 160 | 2,069 | 1,416 | 91.8% | 59.4% | 92.8% | 41.0% | 78 | 14 | 29.4 | 819.9 | Below primary sensitivity criterion |
| R188 | 22.5 | 8 | SPV ≤4°/s | 1,835 | 117 | 1,870 | 1,615 | 94.0% | 53.7% | 94.1% | 36.5% | 52 | 9 | 21.5 | 730.9 | Below primary sensitivity criterion |
| R189 | 22.5 | 10 | SPV ≤4°/s | 1,861 | 91 | 1,681 | 1,804 | 95.3% | 48.2% | 94.9% | 32.6% | 39 | 6 | 16.7 | 651.8 | Below primary sensitivity criterion |
| R190 | 22.5 | 11 | SPV ≤4°/s | 1,871 | 81 | 1,589 | 1,896 | 95.9% | 45.6% | 95.1% | 30.7% | 35 | 5 | 14.9 | 614.3 | Below primary sensitivity criterion |
| R191 | 25 | 5 | SPV ≤4°/s | 1,740 | 212 | 2,290 | 1,195 | 89.1% | 65.7% | 91.5% | 46.0% | 100 | 18 | 39.0 | 920.4 | Below primary sensitivity criterion |
| R192 | 25 | 6 | SPV ≤4°/s | 1,761 | 191 | 2,196 | 1,289 | 90.2% | 63.0% | 92.0% | 43.9% | 88 | 16 | 35.1 | 878.1 | Below primary sensitivity criterion |
| R193 | 25 | 8 | SPV ≤4°/s | 1,809 | 143 | 1,984 | 1,501 | 92.7% | 56.9% | 93.3% | 39.1% | 61 | 11 | 26.3 | 782.4 | Below primary sensitivity criterion |
| R194 | 25 | 10 | SPV ≤4°/s | 1,836 | 116 | 1,789 | 1,696 | 94.1% | 51.3% | 93.9% | 35.0% | 48 | 8 | 21.3 | 700.8 | Below primary sensitivity criterion |
| R195 | 25 | 11 | SPV ≤4°/s | 1,849 | 103 | 1,688 | 1,797 | 94.7% | 48.4% | 94.2% | 32.9% | 43 | 7 | 18.9 | 658.8 | Below primary sensitivity criterion |
| R196 | 30 | 5 | SPV ≤4°/s | 1,625 | 327 | 2,573 | 912 | 83.2% | 73.8% | 88.7% | 53.3% | 166 | 34 | 60.1 | 1066.8 | Below primary sensitivity criterion |
| R197 | 30 | 6 | SPV ≤4°/s | 1,656 | 296 | 2,446 | 1,039 | 84.8% | 70.2% | 89.2% | 50.4% | 147 | 29 | 54.4 | 1008.6 | Below primary sensitivity criterion |
| R198 | 30 | 8 | SPV ≤4°/s | 1,721 | 231 | 2,210 | 1,275 | 88.2% | 63.4% | 90.5% | 44.9% | 108 | 19 | 42.5 | 897.9 | Below primary sensitivity criterion |
| R199 | 30 | 10 | SPV ≤4°/s | 1,767 | 185 | 1,986 | 1,499 | 90.5% | 57.0% | 91.5% | 39.9% | 79 | 14 | 34.0 | 798.6 | Below primary sensitivity criterion |
| R200 | 30 | 11 | SPV ≤4°/s | 1,784 | 168 | 1,864 | 1,621 | 91.4% | 53.5% | 91.7% | 37.4% | 74 | 13 | 30.9 | 747.5 | Below primary sensitivity criterion |

*Exact confidence intervals and the complete metric set are available in the companion Excel workbook. All 200 rows are distinct parameter combinations.*

Supplementary Table S3. Sensitivity analysis across alternative CP reference thresholds

| **Cohort** | **CP reference threshold (%)** | **CP positive n** | **CP negative n** | **TP/FN/TN/FP** | **Sensitivity (95% CI)** | **Specificity (95% CI)** | **NPV (95% CI)** | **LR− (95% CI)** | **Candidate stop rate (95% CI)** |
| --- | --- | --- | --- | --- | --- | --- | --- | --- | --- |
| Derivation cohort | ≥20 | 2,525 | 2,912 | 2,405/120/1,207/1,705 | 95.2% (94.3%–96.0%) | 41.4% (39.7%–43.3%) | 91.0% (89.3%–92.4%) | 0.115 (0.096–0.137) | 24.4% (23.3%–25.6%) |
| Derivation cohort | ≥25 | 1,952 | 3,485 | 1,906/46/1,281/2,204 | 97.6% (96.9%–98.3%) | 36.8% (35.2%–38.4%) | 96.5% (95.4%–97.5%) | 0.064 (0.048–0.086) | 24.4% (23.3%–25.6%) |
| Derivation cohort | ≥30 | 1,522 | 3,915 | 1,501/21/1,306/2,609 | 98.6% (97.9%–99.1%) | 33.4% (31.9%–34.9%) | 98.4% (97.6%–99.0%) | 0.041 (0.027–0.063) | 24.4% (23.3%–25.6%) |
| Derivation cohort | ≥40 | 955 | 4,482 | 953/2/1,325/3,157 | 99.8% (99.2%–100.0%) | 29.6% (28.2%–30.9%) | 99.8% (99.5%–100.0%) | 0.007 (0.002–0.028) | 24.4% (23.3%–25.6%) |
| Temporal validation cohort | ≥20 | 1,098 | 1,218 | 1,045/53/494/724 | 95.2% (93.7%–96.4%) | 40.6% (37.8%–43.4%) | 90.3% (87.5%–92.7%) | 0.119 (0.091–0.156) | 23.6% (21.9%–25.4%) |
| Temporal validation cohort | ≥25 | 846 | 1,470 | 832/14/533/937 | 98.3% (97.2%–99.1%) | 36.3% (33.8%–38.8%) | 97.4% (95.7%–98.6%) | 0.046 (0.027–0.077) | 23.6% (21.9%–25.4%) |
| Temporal validation cohort | ≥30 | 663 | 1,653 | 659/4/543/1,110 | 99.4% (98.5%–99.8%) | 32.8% (30.6%–35.2%) | 99.3% (98.1%–99.8%) | 0.018 (0.007–0.049) | 23.6% (21.9%–25.4%) |
| Temporal validation cohort | ≥40 | 439 | 1,877 | 439/0/547/1,330 | 100.0% (99.2%–100.0%) | 29.1% (27.1%–31.3%) | 100.0% (99.3%–100.0%) | 0.000 (0.000–0.062) | 23.6% (21.9%–25.4%) |

*The final composite rule was applied unchanged; no threshold was reselected for an alternative CP endpoint.*

Supplementary Table S4. Representative warm monothermal candidate rules meeting the prespecified sensitivity criterion

| Rule ID | Spontaneous nystagmus condition | MCA threshold (%) | Minimum warm SPV threshold (°/s) | Sensitivity (95% CI) | Specificity (95% CI) | NPV (95% CI) | FN | Candidate stop rate | CP ≥30% FN | CP ≥40% FN | Selection description |
| --- | --- | --- | --- | --- | --- | --- | --- | --- | --- | --- | --- |
| R085 | SPV <2°/s | <20 | >11 | 97.6% (96.9%–98.3%) | 34.3% (32.7%–35.9%) | 96.3% (95.1%–97.3%) | 46 | 22.8% | 22 | 4 | Specificity-first rule for this spontaneous nystagmus threshold |
| R130 | SPV <4°/s | <17.5 | >11 | 97.6% (96.9%–98.3%) | 36.8% (35.2%–38.4%) | 96.5% (95.4%–97.5%) | 46 | 24.4% | 21 | 2 | Final selected rule |
| R027 | SPV = 0°/s | <17.5 | >6 | 97.7% (97.0%–98.4%) | 25.3% (23.8%–26.8%) | 95.2% (93.7%–96.5%) | 44 | 17.0% | 19 | 3 | Specificity-first rule for this spontaneous nystagmus threshold |
| R180 | SPV ≤4°/s | <17.5 | >11 | 97.6% (96.8%–98.2%) | 37.1% (35.5%–38.7%) | 96.5% (95.4%–97.4%) | 47 | 24.6% | 21 | 2 | Specificity-first rule for this spontaneous nystagmus threshold |

*All rules were generated exclusively in the derivation cohort. The complete 200-rule grid is reported in Supplementary Table S2.*

Supplementary Table S5. Two-by-two classification table of the final warm composite rule in the temporal validation cohort

| Rule disposition | CP ≥25% | CP <25% | Total |
| --- | --- | --- | --- |
| Complete bithermal testing | 832 | 937 | 1,769 |
| Candidate stop | 14 | 533 | 547 |
| Total | 846 | 1,470 | 2,316 |

*Test-positive classification was defined as requiring complete bithermal testing; candidate stop was the index-test-negative classification.*

Supplementary Table S6. Estimated clinical efficiency of the final warm composite rule

| Cohort | n | Candidate stop, n (%) | Complete bithermal testing, n | Missed CP ≥25% per 1,000 (95% CI) | Missed CP ≥30% per 1,000 (95% CI) | Missed CP ≥40% per 1,000 (95% CI) | Avoided cold irrigations per 1,000 (95% CI) | Reduction in planned cold irrigations | Reduction in all caloric irrigations |
| --- | --- | --- | --- | --- | --- | --- | --- | --- | --- |
| Derivation cohort | 5,437 | 1,327 (24.4%) | 4,110 | 8.5 (6.1–10.9) | 3.9 (2.4–5.7) | 0.4 (0.0–0.9) | 488.1 (463.9–512.0) | 24.4% | 12.2% |
| Temporal validation cohort | 2,316 | 547 (23.6%) | 1,769 | 6.0 (3.0–9.5) | 1.7 (0.4–3.5) | 0.0 (0.0–0.0) | 472.4 (437.8–507.8) | 23.6% | 11.8% |

*Confidence intervals for per-1,000 measures were obtained from 2,000 patient-level bootstrap resamples. The observed CP ≥40% burden was zero; the bootstrap interval is supplemented by the exact zero-event bound in Table 4 and Figure 4.*

Supplementary Table S7. Performance of warm and cold monothermal testing across directional preponderance strata

| Absolute DP stratum | Scheme | n | CP positive/negative, n | AUC (95% CI) | Sensitivity (95% CI) | Specificity (95% CI) | NPV (95% CI) | False-negative rate (95% CI) |
| --- | --- | --- | --- | --- | --- | --- | --- | --- |
| <10% | Warm | 868 | 229/639 | 0.971 (0.962–0.980) | 100.0% (98.4%–100.0%) | 24.1% (20.8%–27.6%) | 100.0% (97.6%–100.0%) | 0.0% (0.0%–1.6%) |
| <10% | Cold | 868 | 229/639 | 0.965 (0.954–0.975) | 100.0% (98.4%–100.0%) | 23.2% (19.9%–26.6%) | 100.0% (97.5%–100.0%) | 0.0% (0.0%–1.6%) |
| 10 to <20% | Warm | 677 | 246/431 | 0.922 (0.897–0.942) | 100.0% (98.5%–100.0%) | 13.0% (10.0%–16.5%) | 100.0% (93.6%–100.0%) | 0.0% (0.0%–1.5%) |
| 10 to <20% | Cold | 677 | 246/431 | 0.863 (0.836–0.890) | 100.0% (98.5%–100.0%) | 21.3% (17.6%–25.5%) | 100.0% (96.1%–100.0%) | 0.0% (0.0%–1.5%) |
| 20 to <30% | Warm | 437 | 193/244 | 0.869 (0.823–0.908) | 97.4% (94.1%–99.2%) | 7.8% (4.8%–11.9%) | 79.2% (57.8%–92.9%) | 2.6% (0.8%–5.9%) |
| 20 to <30% | Cold | 437 | 193/244 | 0.662 (0.608–0.716) | 91.2% (86.3%–94.8%) | 11.5% (7.8%–16.2%) | 62.2% (46.5%–76.2%) | 8.8% (5.2%–13.7%) |
| ≥30% | Warm | 334 | 178/156 | 0.849 (0.801–0.892) | 97.8% (94.3%–99.4%) | 0.0% (0.0%–2.3%) | 0.0% (0.0%–60.2%) | 2.2% (0.6%–5.7%) |
| ≥30% | Cold | 334 | 178/156 | 0.369 (0.311–0.425) | 84.3% (78.1%–89.3%) | 0.0% (0.0%–2.3%) | 0.0% (0.0%–12.3%) | 15.7% (10.7%–21.9%) |

*Diagnostic proportions were calculated at the prespecified 5% single-MCA benchmark. LR values were omitted from strata in which a zero cell made them not estimable.*

Supplementary Table S8. Performance of warm and cold monothermal testing across spontaneous nystagmus strata

| Spontaneous nystagmus stratum | Scheme | n | CP positive/negative, n | AUC (95% CI) | Sensitivity (95% CI) | Specificity (95% CI) | NPV (95% CI) | False-negative rate (95% CI) |
| --- | --- | --- | --- | --- | --- | --- | --- | --- |
| 0°/s | Warm | 881 | 268/613 | 0.890 (0.867–0.911) | 99.6% (97.9%–100.0%) | 16.6% (13.8%–19.8%) | 99.0% (94.7%–100.0%) | 0.4% (0.0%–2.1%) |
| 0°/s | Cold | 881 | 268/613 | 0.802 (0.770–0.832) | 97.0% (94.2%–98.7%) | 18.9% (15.9%–22.3%) | 93.5% (87.7%–97.2%) | 3.0% (1.3%–5.8%) |
| >0 to <2°/s | Warm | 901 | 342/559 | 0.905 (0.883–0.926) | 98.8% (97.0%–99.7%) | 16.5% (13.5%–19.8%) | 95.8% (89.7%–98.9%) | 1.2% (0.3%–3.0%) |
| >0 to <2°/s | Cold | 901 | 342/559 | 0.765 (0.730–0.799) | 92.4% (89.1%–95.0%) | 18.1% (15.0%–21.5%) | 79.5% (71.5%–86.2%) | 7.6% (5.0%–10.9%) |
| 2 to <4°/s | Warm | 439 | 190/249 | 0.873 (0.834–0.907) | 97.9% (94.7%–99.4%) | 12.0% (8.3%–16.8%) | 88.2% (72.5%–96.7%) | 2.1% (0.6%–5.3%) |
| 2 to <4°/s | Cold | 439 | 190/249 | 0.800 (0.757–0.842) | 96.3% (92.6%–98.5%) | 18.1% (13.5%–23.4%) | 86.5% (74.2%–94.4%) | 3.7% (1.5%–7.4%) |
| ≥4°/s | Warm | 95 | 46/49 | 0.765 (0.659–0.856) | 100.0% (92.3%–100.0%) | 10.2% (3.4%–22.2%) | 100.0% (47.8%–100.0%) | 0.0% (0.0%–7.7%) |
| ≥4°/s | Cold | 95 | 46/49 | 0.522 (0.408–0.632) | 91.3% (79.2%–97.6%) | 12.2% (4.6%–24.8%) | 60.0% (26.2%–87.8%) | 8.7% (2.4%–20.8%) |

*Point estimates of 100% should be interpreted with event counts and confidence intervals.*

Supplementary Table S9. Exploratory cross-temperature schemes

| Scheme | Formula | k | Selected threshold (%) | Validation AUC (95% CI) | Sensitivity (95% CI) | Specificity (95% CI) | NPV (95% CI) | FN | Candidate stop rate |
| --- | --- | --- | --- | --- | --- | --- | --- | --- | --- |
| RW + LC | \|(RW − k×LC)/(RW + k×LC)\|×100% | 1.287 | 5 | 0.833 (0.816–0.851) | 96.8% (95.4%–97.9%) | 16.9% (15.0%–18.9%) | 90.2% (86.0%–93.4%) | 27 | 11.9% |
| LW + RC | \|(LW − k×RC)/(LW + k×RC)\|×100% | 1.287 | 5 | 0.826 (0.808–0.845) | 97.3% (95.9%–98.3%) | 15.3% (13.5%–17.2%) | 90.7% (86.4%–94.0%) | 23 | 10.7% |

*The coefficient was estimated only in the derivation subgroup with CP <20%, DP <10%, and spontaneous nystagmus SPV = 0 °/s. Temperature and ear side are fully confounded.*

Supplementary Table S10. Individual characteristics and threshold proximity of false-negative cases in the temporal validation cohort

Panel A Reference-standard results and monothermal asymmetry

| Case | CP (%) | CP severity | CP weaker side | DP (%) | DP direction | \|MCA_W\| (%) | \|MCA_C\| (%) |
| --- | --- | --- | --- | --- | --- | --- | --- |
| FN01 | 25.4 | 25 to <30% | Left | 16.4 | Right-beating predominance | 9.1 | 41.2 |
| FN02 | 25.6 | 25 to <30% | Right | 15.4 | Left-beating predominance | 10.0 | 42.1 |
| FN03 | 26.0 | 25 to <30% | Right | 1.4 | Left-beating predominance | 17.0 | 50.0 |
| FN04 | 26.1 | 25 to <30% | Left | 17.4 | Right-beating predominance | 7.1 | 55.6 |
| FN05 | 26.3 | 25 to <30% | Right | 5.3 | Left-beating predominance | 16.7 | 42.9 |
| FN06 | 26.9 | 25 to <30% | Right | 11.5 | Left-beating predominance | 14.3 | 41.7 |
| FN07 | 27.3 | 25 to <30% | Left | 21.2 | Right-beating predominance | 5.3 | 57.1 |
| FN08 | 27.7 | 25 to <30% | Right | 9.2 | Left-beating predominance | 14.6 | 50.0 |
| FN09 | 28.4 | 25 to <30% | Right | 23.5 | Left-beating predominance | 7.7 | 38.2 |
| FN10 | 28.6 | 25 to <30% | Left | 11.9 | Right-beating predominance | 14.9 | 45.9 |
| FN11 | 31.4 | 30 to <40% | Left | 20.0 | Right-beating predominance | 10.5 | 56.2 |
| FN12 | 34.7 | 30 to <40% | Left | 34.7 | Right-beating predominance | 0.0 | 73.9 |
| FN13 | 37.7 | 30 to <40% | Right | 24.6 | Left-beating predominance | 11.1 | 76.0 |
| FN14 | 37.9 | 30 to <40% | Right | 26.4 | Left-beating predominance | 11.6 | 63.6 |

Panel B Caloric responses and spontaneous nystagmus

| Case | RW (°/s) | LW (°/s) | RC (°/s) | LC (°/s) | WarmMin (°/s) | Spontaneous nystagmus direction | Spontaneous nystagmus SPV (°/s) |
| --- | --- | --- | --- | --- | --- | --- | --- |
| FN01 | 18.0 | 15.0 | 24.0 | 10.0 | 15.0 | Left-beating; downbeat | 2.0 |
| FN02 | 18.0 | 22.0 | 11.0 | 27.0 | 18.0 | Left-beating | 2.0 |
| FN03 | 22.0 | 31.0 | 5.0 | 15.0 | 22.0 | None | 0.0 |
| FN04 | 15.0 | 13.0 | 14.0 | 4.0 | 13.0 | Left-beating | 1.0 |
| FN05 | 15.0 | 21.0 | 6.0 | 15.0 | 15.0 | Upbeat | 2.0 |
| FN06 | 12.0 | 16.0 | 7.0 | 17.0 | 12.0 | Upbeat | 1.0 |
| FN07 | 20.0 | 18.0 | 22.0 | 6.0 | 18.0 | Left-beating | 2.0 |
| FN08 | 38.0 | 51.0 | 13.0 | 39.0 | 38.0 | None | 0.0 |
| FN09 | 12.0 | 14.0 | 17.0 | 38.0 | 12.0 | Not recorded | 0.0 |
| FN10 | 54.0 | 40.0 | 54.0 | 20.0 | 40.0 | Not recorded | 0.0 |
| FN11 | 21.0 | 17.0 | 25.0 | 7.0 | 17.0 | Left-beating | 1.0 |
| FN12 | 13.0 | 13.0 | 20.0 | 3.0 | 13.0 | Left-beating | 2.0 |
| FN13 | 16.0 | 20.0 | 3.0 | 22.0 | 16.0 | Upbeat; right-beating | 2.0 |
| FN14 | 19.0 | 24.0 | 8.0 | 36.0 | 19.0 | None | 0.0 |

Panel C Distance from the candidate-stop thresholds

| Case | CP − 25 (percentage points) | 17.5 − \|MCA_W\| (percentage points) | WarmMin − 11 (°/s) | 4 − spontaneous nystagmus SPV (°/s) |
| --- | --- | --- | --- | --- |
| FN01 | 0.4 | 8.4 | 4.0 | 2.0 |
| FN02 | 0.6 | 7.5 | 7.0 | 2.0 |
| FN03 | 1.0 | 0.5 | 11.0 | 4.0 |
| FN04 | 1.1 | 10.4 | 2.0 | 3.0 |
| FN05 | 1.3 | 0.8 | 4.0 | 2.0 |
| FN06 | 1.9 | 3.2 | 1.0 | 3.0 |
| FN07 | 2.3 | 12.2 | 7.0 | 2.0 |
| FN08 | 2.7 | 2.9 | 27.0 | 4.0 |
| FN09 | 3.4 | 9.8 | 1.0 | 4.0 |
| FN10 | 3.6 | 2.6 | 29.0 | 4.0 |
| FN11 | 6.4 | 7.0 | 6.0 | 3.0 |
| FN12 | 9.7 | 17.5 | 2.0 | 2.0 |
| FN13 | 12.7 | 6.4 | 5.0 | 2.0 |
| FN14 | 12.9 | 5.9 | 8.0 | 4.0 |

*Note. For 17.5 − |MCA_W|, WarmMin − 11, and 4 − spontaneous nystagmus SPV, positive values indicate that the corresponding measure remained within the candidate-stop range; for CP − 25, a positive value indicates the amount by which CP exceeded the abnormality threshold. The candidate-stop rule was |MCA_W| <17.5%, WarmMin >11°/s, and spontaneous nystagmus SPV <4°/s. Unrecorded spontaneous nystagmus was analyzed as 0°/s according to the prespecified center reporting convention; its direction is shown as Not recorded. CP, canal paresis; DP, directional preponderance; LW, left warm; LC, left cold; MCA, monothermal caloric asymmetry; RC, right cold; RW, right warm; SPV, slow-phase velocity.*
